# A High Spatial and Depth Resolution Deep-UV 266 nm Wavelength Laser-Based Integrated LIBS, Fluorescence, and Raman System for Probing Lunar and Planetary Simulants and Geological Materials

**DOI:** 10.1021/acsomega.5c02748

**Published:** 2025-08-15

**Authors:** Anil Aryal, Pawan K. Kanaujia, Atchutananda Surampudi, Dina M. Bower, Tilak Hewagama, Narasimha S. Prasad, William B. Moore, Mool C. Gupta

**Affiliations:** † Charles L. Brown Department of Electrical and Computer Engineering, 744402 University of Virginia, Charlottesville, Virginia 22904, United States; ‡ Laser and Plasma Technologies, Charlottesville, Virginia 22903, United States; § Department of Astronomy, 1068 University of Maryland, College Park, Maryland 20742, United States; ∥ 53523 NASA Goddard Space Flight Center, Greenbelt, Maryland 20771, United States; ⊥ 53524 NASA Langley Research Center, Hampton, Virginia 23681, United States; # Department of Atmospheric & Planetary Sciences, 3726 Hampton University, Hampton, Virginia 23668, United States

## Abstract

A high spatial and depth resolution deep-ultraviolet (UV) 266 nm wavelength laser-based integrated laser-induced breakdown spectroscopy (LIBS), fluorescence, and Raman (LFR) system was developed for qualitatively probing lunar and planetary simulants and geological materials. The LFR system involves the use of a simple, low-weight, and compact LFR *optical head* (6 cm × 3 cm × 5 cm) consisting of common optics (filters, lens, and optical fibers) while being supported by an external *single* deep-UV pulsed laser source at 266 nm wavelength and a shared spectrometer for multifunctional Raman, fluorescence, and LIBS spectroscopy. A spatial resolution of about 15 μm was achieved and can be further decreased to under a μm. The submicron depth resolution was achieved by the 266 nm wavelength laser ablation process and can be further improved to tens of nanometers. The performance of the integrated LFR system was evaluated by probing both standard materials (organic and inorganic) as well as previously characterized simulants and geological materials, which predominantly consisted of mixtures of silicates and oxides of various metals. The obtained LIBS spectra agreed well with the literature, and no significant spatial and depth variation in composition was noted. The optical head was correctly able to obtain good Raman signatures from a range of materials, including organic (alcohol, alkanes, amino acids, and polymer) and inorganic species (sulfates, carbonates), such as water, selenite, and calcite. Analysis of the LIBS spectra correctly revealed the presence of major elements, such as Mg, Si, Fe, Al, Ca, Mn, and Ti, in the basalt, gneiss, meteorite, and simulants. This work establishes a foundation for developing single laser-spectrometer-based compact multifunctional deep-UV spectroscopic systems performing both Raman and LIBS alongside fluorescence, paving the way for further advancements toward miniaturization.

## Graphical abstract

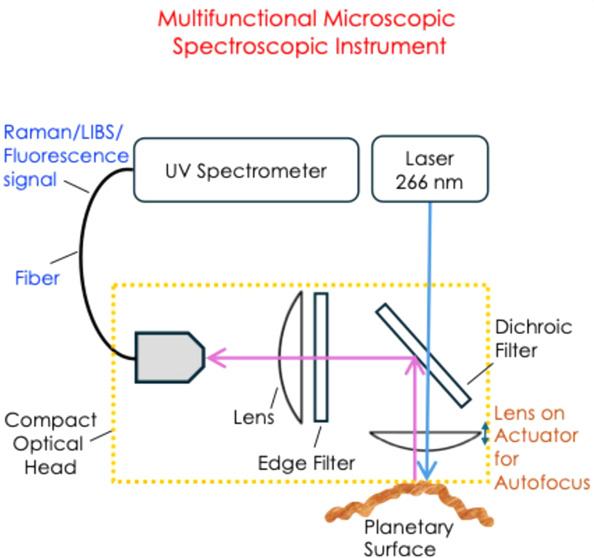

## 1 Introduction

In the pursuit of gaining a deeper understanding of the origin and evolution of our solar system, as well as the search for signs of habitability and potential life beyond Earth, various space missions (landers, rovers, and orbiters) have been deployed in the past and recent years.^1–4^ These missions have played a pivotal role in expanding our knowledge and pushing the boundaries of exploration, leading to groundbreaking discoveries in the fields of planetary science, habitability studies, and the quest for extraterrestrial life. A considerable portion of these investigations has been focused on two main categories of celestial bodies: icy bodies such as Enceladus and Europa and rocky bodies such as Mars, asteroids, and moons.

Laser-based spectroscopic techniques such as Raman and laser-induced breakdown spectroscopy (LIBS) have proven to be viable methods for the in situ exploration of planetary bodies. Raman spectroscopy provides an invaluable and nondestructive analytical tool for molecular fingerprinting and monitoring changes in molecular bond structure. LIBS enables direct solid sampling to provide in situ, real-time elemental detection and identification of target materials. Together, LIBS, fluorescence, and Raman spectroscopy (LFR) comprehensive measurements can reveal the chemical structure and elemental composition of organic molecules, minerals, and salts.^5^

NASA has already implemented the spectroscopy probe ChemCam in the Mars Science Laboratory (MSL) mission in 2012 to acquire LIBS measurements at a range of several meters.^6^ Part of the current Mars 2020 Perseverance Rover payload utilizes SuperCam (a combined LIBS-Raman instrument) and SHERLOC (a Raman-fluorescence instrument) to enable the identification of elements, organics, and minerals.^7–9^ Although these spectroscopic probes utilize state-of-the-art technologies, the key challenge still exists in miniaturizing the system to reduce power consumption and payload cost. Thus, the continuous development of versatile, low-power, compact, integrated technologies capable of in situ measurements is necessary for the current and future exploration of planetary bodies in our solar system.

While visible to near-IR wavelengths (400–1064 nm) is the most common choice of wavelengths for the LIBS-Raman experiment, the use of shorter (266 nm) wavelength sources for these techniques has been very limited, specifically for the study of extraterrestrial material and its analogue. A few groups have used a deep-ultraviolet (UV) 266 nm laser for LIBS for analysis of nuclear waste, hazardous material (chemical and biological) detection, and geochemical and environmental analysis.^10–15^ Nobody to date has incorporated multifunctional capabilities of Raman, LIBS, and fluorescence with a single deep-UV system. Early instruments on the Mars 2020 mission were deep-UV Raman fluorescence systems utilizing 248.6 nm wavelength to detect organic compounds and minerals on Mars, however, excluding the capability of LIBS. The use of a UV excitation source has the following advantages: the intensity of the Raman signal is expected to increase greatly with a decrease in wavelength (due to 1/λ^4^ dependence), and the signal-interfering fluorescence is suppressed.^16–18^ Similarly, for LIBS, the use of a deep-UV source has advantages in ablation efficiency due to smaller absorption depth in materials,^19^ smaller spot sizes, better spectral repeatability, and higher penetration into the plasma against its shielding effect.^20–22^ Also, it allows finer depth profiling due to the extremely short absorption depth.

In this study, we present a streamlined and compact experimental setup capable of performing all three (Raman, fluorescence, and LIBS measurements) using a *single* 266 nm deep-UV excitation wavelength. Raman and fluorescence measurements can be performed simultaneously, while LIBS can be conducted at the same location after the Raman and fluorescence measurements have been completed. The designed LFR setup uses a single laser and fiber optics, thereby reducing the system size and weight. Herein, we importantly show the feasibility of a deep-UV 266 nm wavelength excitation source for LIBS measurements of lunar and planetary simulants to identify the elements present in the samples. Additional system characteristics, such as elemental investigation with micron spatial resolution and depth profiling study, are presented. The outcomes of this work will be relevant in the development of combined LFR instruments based on deep-UV sources that can be utilized in existing and future planetary science missions, as well as earth-based geological exploration missions, where mass, volume, and power are constrained.

## 2 Experimental Section

### 2.1 LFR Experimental Setup Design and Integration

Our combined experimental LFR setup integrates a single laser, a single spectrometer, and fiber optics, resulting in reduced system mass, size, and power consumption. The schematic and photograph of the laboratory LFR experimental setup are shown in Figure 1a,b, respectively.

**Figure 1:**
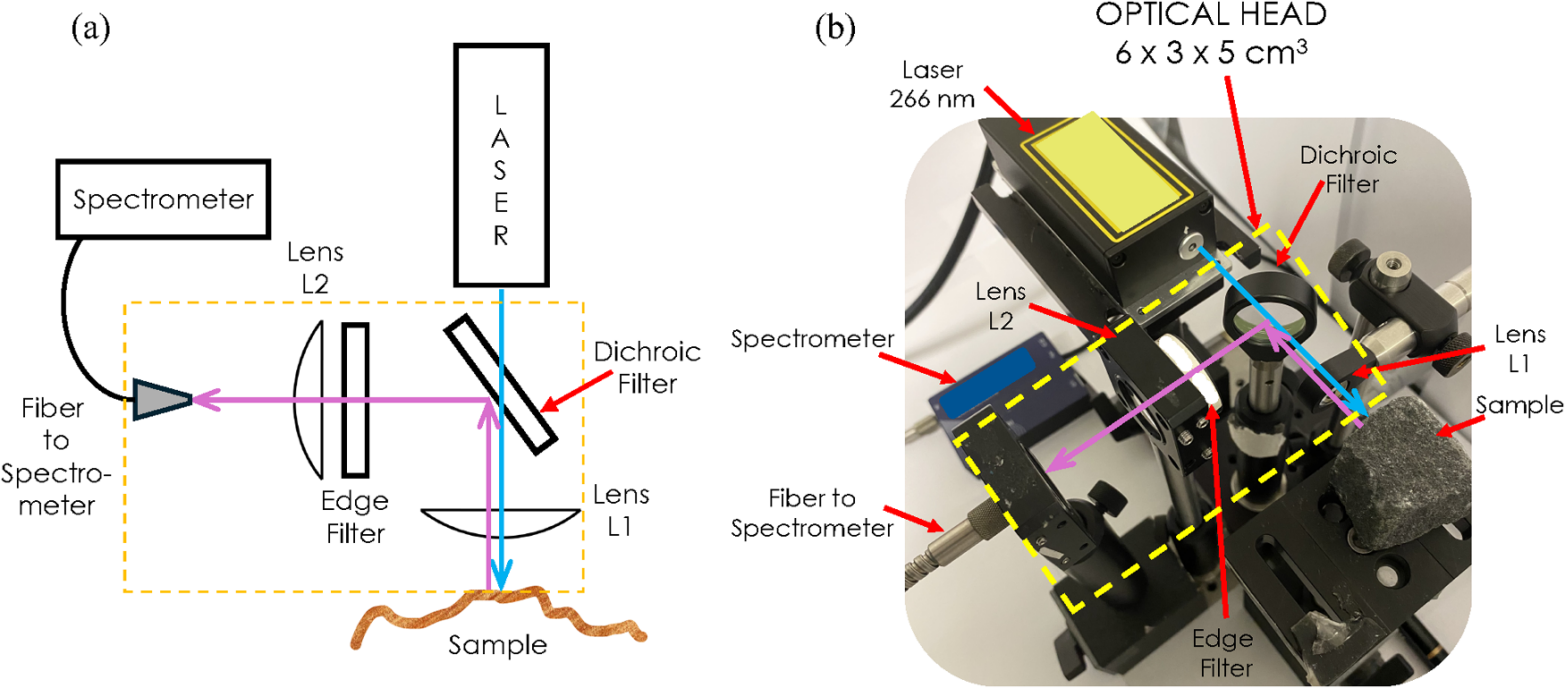
(a) Schematic representation and (b) experimental laboratory setup of the LFR in an on-axis configuration. The compact optical head assembly of 6 cm × 3 cm × 5 cm is shown in the yellow box.

As shown in Figure 1a, the system comprises a 266 nm Nd: YAG Q-switched Microchip laser (from Crylink) was used as a deep-UV excitation source (pulse width: 1.5 ns, frequency: 1 kHz, pulse energy: 12 μJ), a dichroic filter, edge filter, convex lenses L1 and L2, optical fiber, and a compact spectrometer. The laser and collection optics are arranged in a backscattering confocal geometry. The optical head, which consists of the optics needed to collect the spectroscopic information, is depicted in a yellow box in Figure 1b, and it occupies a compact volume of 6 cm × 3 cm × 5 cm, weighing 100 g. While the laser and the spectrometer are considered external to the optical head, they also occupy a compact volume of, respectively, (laser) 4.5 cm × 8.8 cm × 2 cm weighing 400 g and (spectrometer) 7 cm × 7 cm × 1.5 cm weighing 250 g (AvaSpec-Mini 4096 CL from Avantes Inc.).

During the experiment, the laser beam is incident on the sample, leading to an interaction with the material. The emitted signal from the sample is collected by a UV silica convex lens L1 (Thorlabs LA4647) and reflected by the dichroic beam splitter. The dichroic beam splitter for the deep-UV 266 nm system had to be custom-built and is not available commercially, as in the case of Raman spectrometers conventionally operating in the UV/Visible spectrum >380 nm. The authors obtained the filter from a vendor named Omega Filters, who custom-built the filter such that the transmission characteristics show a narrow band-pass at 266 nm when light is incident at an angle of 45°. The reflected signal from the beam splitter passes through the commercially available ultrasteep long-pass edge filter (Semrock RazorEdge LP02-266RU-25, OD > 6), which blocks the 266 nm laser line while allowing the light of a higher wavelength to pass through it. To maximize the signals at the detection end, a UV silica convex lens L2 (*f* = 40 mm, Thorlabs) is utilized to tightly focus the light onto the optical fiber end (600 μm core diameter and 0.22 NA) after passing through the edge filter. The opposite end of the fiber is connected to the compact spectrometer (AvaSpec-Mini 4096 CL from Avantes Inc.). The spectrometer has a 1800 l/mm grating, a 10 μm slit opening, and a spectral resolution of 0.09 nm, capturing spectra within the wavelength range of 200–365 nm. In the spectrometer, a Hamamatsu S13496 (CMOS linear image sensor) is used as a detector. It has a long photosensitive area consisting of 4096 pixels and 7 × 200 μm pixel size. It has a grating efficiency of 60% in the blaze wavelength 250–300 nm. The spectrometer consumes 225 mA, 5 V DC power, weighs 0.175 kg, and has dimensions 6.8 cm × 2 cm × 9.5 cm (*W* × *H* × *L*). Raman, LIBS, and fluorescent spectra were recorded using the “*Avasoft8*” software (from Avantes) and analyzed using the commercial software “*Know it All*”^18^ and “*Atom Analyzer*”.^19^ The fluorescence spectrum of the simulant was recorded using a separate spectrometer from StellarNet, Inc., EPP 2000 CXR-SR-200 with a wider wavelength range (200–1070 nm) and 1 nm resolution. The Raman spectra are generally expressed in relative wavenumbers (in cm^–1^) and range from 0 to 4000 cm^–1^, where 0 cm^–1^ corresponds to the laser wavelength used. For a 266 nm excitation wavelength, the Raman spectra, therefore, range from 266 nm (0 cm^–1^) to approximately 298 nm (4000 cm^–1^). Based on the specifications of the edge filter (cutoff near 268 nm) and dichroic beam splitter, the LFR system is capable of recording Raman spectra from 4800 cm^–1^ down to 280 cm^–1^.

### 2.2 Experimental Calibration

The compact laboratory LFR setup was calibrated by using well-characterized standard materials with known spectral information. For LIBS calibration, silicon (Si) and germanium (Ge) wafers and sheets of aluminum (Al), tin (Sn), and titanium (Ti) were used. Multiple elements were chosen to acquire spectral information from a wide spectral window for better accuracy. Figure S1a–e (in the Supporting Information) shows the LIBS spectra of Si, Sn, Ti, Ge, and Al. The peak detection and the corresponding element identifications were performed using the software “Atom Analyzer.” The measured elemental peak positions are in excellent agreement with the NIST database^23^ with a low error of 0.05 ± 0.03 nm (see Figure S1f and data in Table S1 in the Supporting Information).^23^

The Raman measurements were performed on a variety of organic and inorganic materials, including alcohols, alkanes, amino acids, polymers, sulfates, carbonates, water, selenite, and calcite. To ensure the reliability of the Raman measurements, a systematic calibration was performed by measuring the Raman spectra of standard samples, which demonstrated a good match with the standard sample database.

Figure S2 (in Supporting Information) shows representative Raman spectra of isopropyl alcohol (IPA), along with corresponding spectra obtained from the software “*Know It All*.” These spectra serve as typical examples illustrating the reliability of the measurements. Figure 2 and Supporting Figure S3 showcase the Raman measurements conducted on organic and inorganic materials.

**Figure 2:**
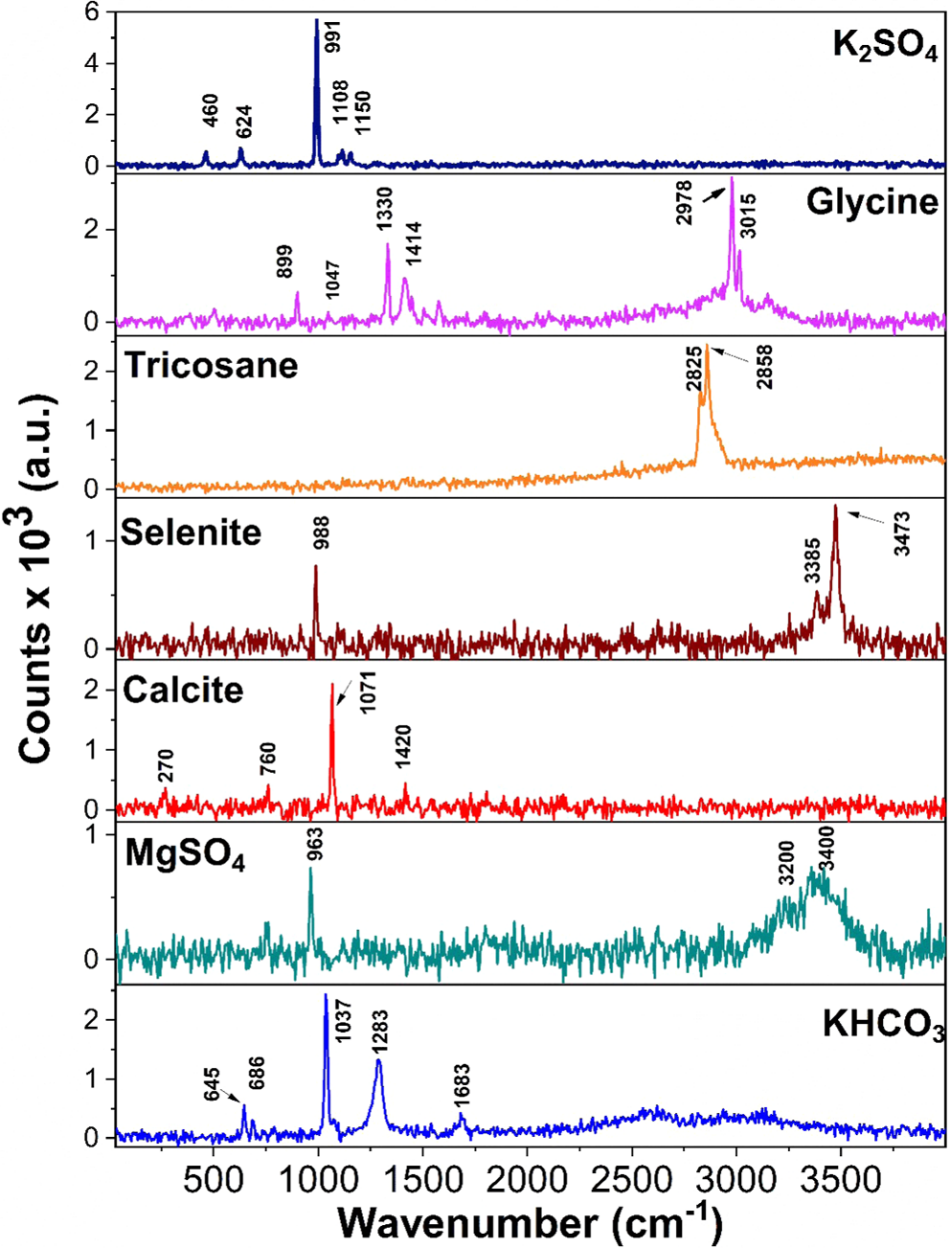
Raman spectra of various solid materials: potassium sulfate (K_2_SO_4_), glycine, tricosane, selenite, calcite, magnesium sulfate (MgSO_4_), and potassium hydrogen carbonate (KHCO_3_).

Fluorescence measurements were conducted on toluene and polystyrene using the LFR system and using the AvaSpec-Mini 4096 CL spectrometer. As depicted in Figure S4 (in Supporting Information), notable emission peaks at 284 nm for toluene and around 320 nm for polystyrene were observed under an excitation wavelength of 266 nm. The fluorescence spectra observed align well with previous findings in the ultraviolet (UV) range, confirming the consistency of our results with existing literature.^24–26^

### 2.3 Samples

Geological field samples relevant to rocky solar system bodies (Iceland basalt, Pacific NW basalt, and gneiss), lunar simulants (Johnson Space Center (JSC1)), (Colorado School of Mines Lunar Mare Type (CSM-LMT1), NASA USGS Lunar Highlands Type (NU-LHT4M), Off Planet Research Lunar Highland Simulant (OPRH2W30)), a planetary simulant (Johnson Space Center- Rock Nest (JSC-RN)), and a meteorite (CR chondrite MIL 090657) were investigated in this study. The geological samples were obtained from NASA’s Goddard Space Flight Center in Maryland, while the simulants and meteorite were sourced from the Johnson Space Center in Houston. Figure S5 displays the optical images of these samples. From the chemical perspective, they predominantly consist of mixtures of silicates and oxides of various metals. Table 1 summarizes the data for the major constituent oxides present in basalt rocks and simulants.^27,28^ More detailed information on the design, mineralogy, chemistry, mechanical, and physical properties of simulants can be found in the planetary simulant database.^28^ The CR chondrite meteorite has been analyzed for the composition of its chondrules and olivine in ref 29.

**Table 1:** Bulk Chemistry of the Basalt Rocks and Simulants in the Form of Oxide wt % ^27,28,30^

	SiO_2_	TiO_2_	Al_2_O_3_	Fe_2_O_3_	MnO	MgO	FeO	CaO	Na_2_O	K_2_O	P_2_O_5_	Cr_2_O_3_	Cl
basalt	49.9	2.73	13.5		0.17	7.23	11.18	11.4	2.22				
JSC-1	47.71	1.59	15.02	3.44	0.18	9.01	7.35	10.42	2.7	0.82	0.66	0.04	0.43
JSC-RN	45.82	1.06	13.66		0.18	9.51	17.71	7.97	3.05	0.54	0.16	0.06	
NU-LHT	46.7	0.41	24.4	4.16	0.07	7.9		13.6	1.26	0.08	0.15		
CSM LMT1	46.9	5.12	15.1			4.11	14	12.2	2.7				
OPRH2W30	47.71	1.59	15.02	3.44	0.18	9.01	7.35	10.42	2.7	0.82	0.66	0.04	

## 3 Results

### 3.1 Surface Morphology and Elemental Composition

Before LIBS measurement, we conducted a comprehensive analysis of the surface morphology and elemental analysis of representative samples, namely, Iceland basalt and JSC-1. To accomplish this, scanning electron microscopy (SEM) and energy-dispersive spectroscopy (EDS) techniques were employed. The SEM image, EDS elemental mapping, and spectra for Iceland basalt are shown in Figure S6a–c (in Supporting Information). The SEM image vividly depicts a rough, granular, and porous surface morphology of Iceland basalt. The EDS spectrum shows elemental peaks corresponding to O, Si, Al, Mg, Fe, Ca, Na, and Ti, and their corresponding wt % present in Iceland basalt. Furthermore, the EDS mappings indicate a uniform distribution of elements across a 250 × 150 μm area of the sample. The EDS results are well aligned with the oxides commonly reported for basalt rocks (see Table 1). Other geological samples (Pacific NW basalt and gneiss) have shown characteristics similar to those of Iceland basalt in EDS analysis. The elemental analysis of the representative simulant (JSC-1) is provided in Figure S7 (in the Supporting Information), which presents the SEM image, EDS mapping, and the spectrum.

### 3.2 Surface and Subsurface Analysis of Geological Samples, Lunar and Planetary Simulants Using LIBS

Using our experimental system, we performed LIBS experiments on a set of geological field samples, including Iceland basalt, Pacific NW basalt, and gneiss, as well as planetary simulants. The experiments were performed at room temperature and under ambient conditions. During the experiments, the LIBS spectra were collected for 1 s integration time, gradually moving the sample to a new position. The acquired spectra of the samples demonstrated good-quality LIBS spectra, which are shown in Figures 3 and 4.

**Figure 3:**
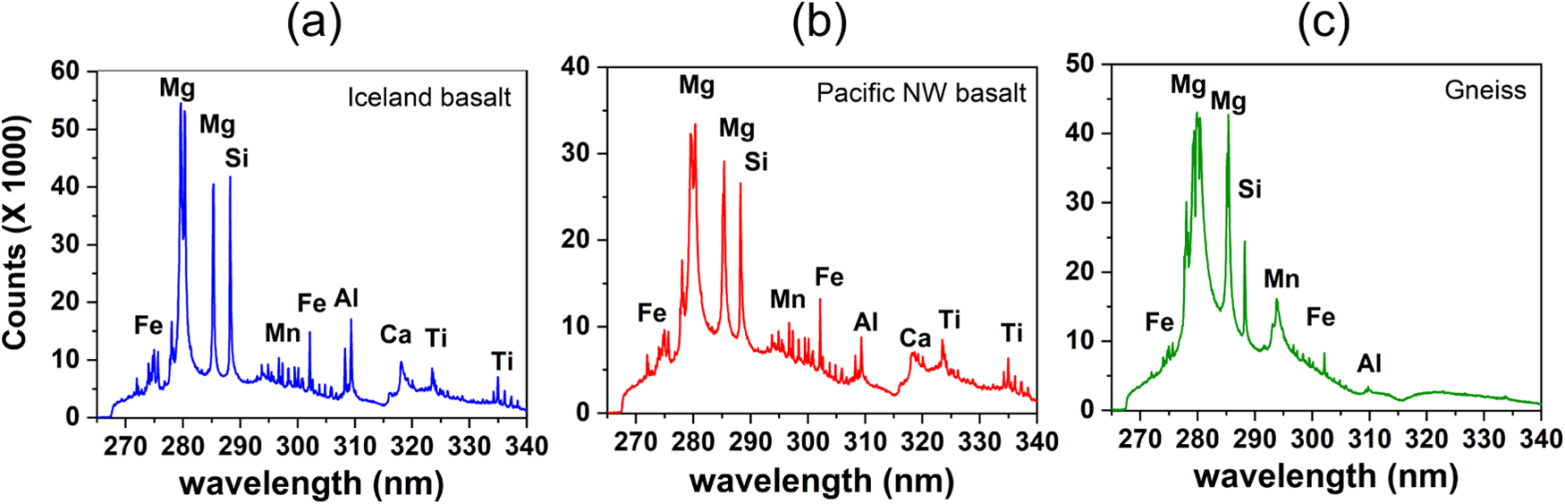
LIBS spectra of (a) Iceland basalt, (b) Pacific NW basalt, and (c) gneiss. The spectra presented are without correction for the continuum emission.

**Figure 4:**
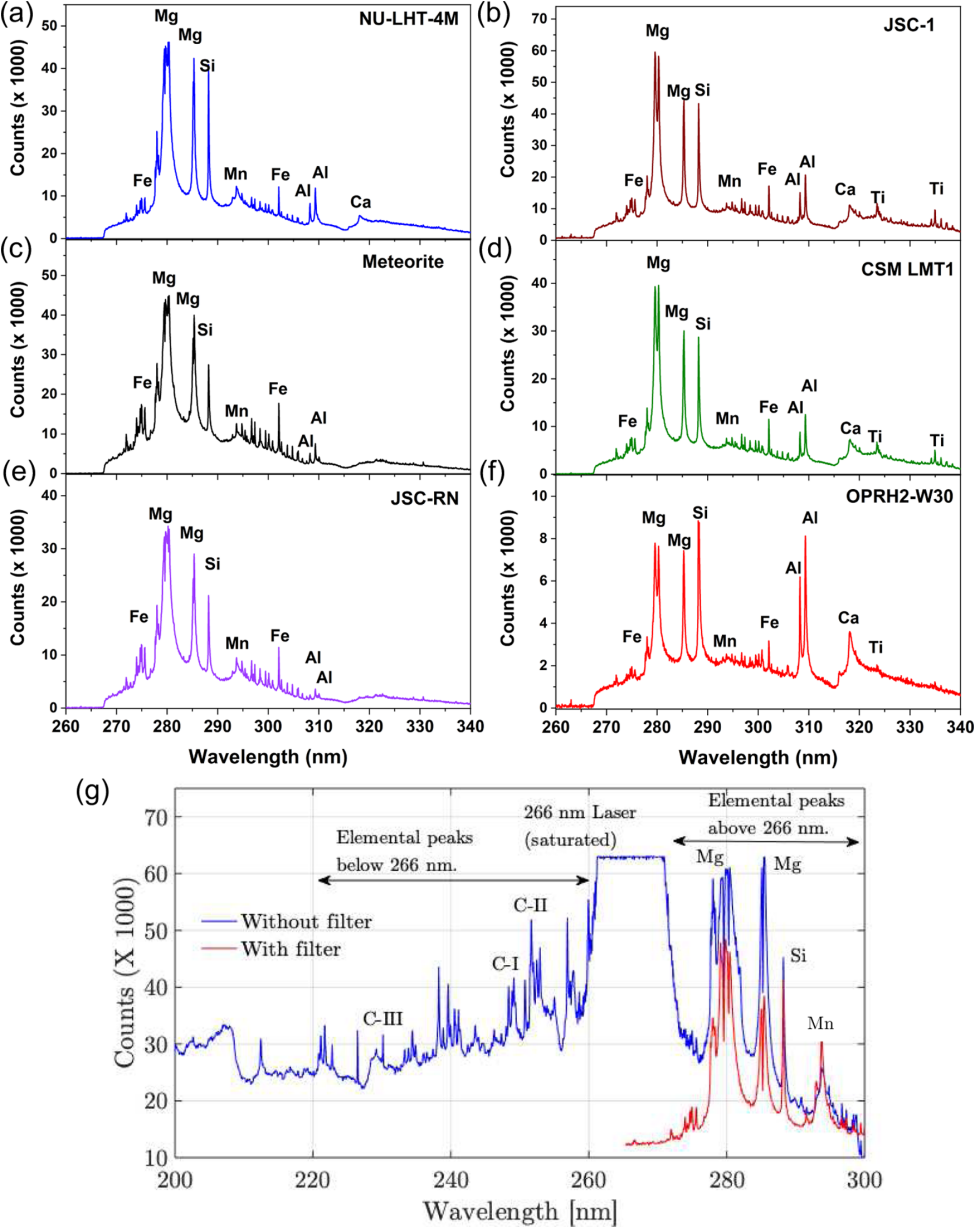
(a–f) LIBS spectra of simulants and a meteorite collected using the current setup based on a 266 nm wavelength, showing the elemental composition. (g) LIBS spectra of gneiss with and without the edge filter, allowing broader spectral range data.

Distinctive peaks observed in the LIBS spectra at specific wavelengths are indicative of the fingerprint characteristics of the elements present in the target material. The major elements identified in these samples included Mg, Si, Fe, Al, Ca, Mn, and Ti. This analysis is complemented by EDS measurements and elemental information obtained for minerals such as olivine, pyroxene, and plagioclase, which are present in these samples.^31^ Results affirm that accurate elemental identification of both geological samples and simulants can be achieved using the laboratory setup based on the 266 nm laser system. To compare our results, we also measured the LIBS spectra using a 1064 nm laser-based setup assembled in the laboratory. The measured results are shown in Figure S16 of the Supporting Information. The results show similar LIBS spectra that match the major elemental peaks, thereby validating our findings. Additionally, these results were also confirmed with data in the literature.^30,32^

To broaden the LIBS spectrum range for elements of interest, such as carbon, the setup can be modified by removing the edge filter and attenuating the laser power (to prevent excessive saturation of the detector). To demonstrate this capability, the edge filter was removed, and laser power was attenuated by 30%, which allowed the obtaining of LIBS peaks below 266 nm, as shown in Figure 4g, for the mineral gneiss. Note that the LIBS peaks greater than 266 nm are still detectable as before, while also allowing the carbon I, II, and III LIBS peaks below 266 nm to be detected.

In addition to probing the elemental composition from the surface, another important feature of LIBS is subsurface analysis through depth profiling. Due to the shallow absorption depth of the 266 nm wavelength in the material and higher ablation efficiency at UV radiation, accurate depth profiling of composition can be expected. We conducted a depth profiling study using a 266 nm wavelength on Si standard and the geological sample (basalt) by gradually ablating the material from the surface and successively recording the LIBS spectra. Interestingly, for a higher number of pulses that correspond to laser repetition rate >50 Hz, LIBS spectra were only observed during the first ablation from the surface, and subsequent laser exposure did not produce the spectra (Figure S8 in the Supporting Information). The absence of LIBS spectra at a higher number of laser pulses is due to the loss of focus with the increasing number of pulses, which results in insufficient laser power to generate the plasma (see the numerical calculations in the Supporting Information). Since successive spectra were not observed at higher pulse numbers, we optimized the laser parameters for a lower number of pulses. The lower number of laser pulses also ensures information is collected from a few layers beneath the surface.

#### 3.2.1. Silicon Sample

For the depth profile study, a crystalline silicon wafer was chosen as a control sample because of its low surface roughness, uniform composition, and good-quality LIBS spectra. The laser repetition rate was optimized to 10 Hz. As shown in Figure S9a (in Supporting Information), a series of laser marks 1 through 7 were ablated on Si. The first mark was made with a set of 10 pulses (shot 1). For the second mark, 10 pulses (shot 1) were fired initially, followed by an additional 10 pulses (shot 2), totaling 20 pulses at that point. This procedure was repeated for other laser marks to ensure the variation in crater depth between the laser marks. The 2D and 3D topographic profile of the crater was obtained using a Hirox RH-8800 light microscope. The 2D images of the craters formed by ablation are shown in Figure S9b–h (in Supporting Information). The images show the material ejected and the area damage around the crater walls with an increasing number of pulses (shots). The melting and resolidification of the materials at the crater edges were observed. The depth of the crater was extracted from a 3D topographic profile (see Figure S10 in the Supporting Information) and was approximately 8 μm for the crater formed by the first set of 10 pulses (i.e., shot 1). The 3D profile of other craters is shown in Figure S11 (in Supporting Information). Interestingly, the crater depth does not show a clear relationship with increasing pulses, as the laser is no longer at focus and has a normal incidence on cone-shaped crater surfaces. Instead of ejecting material and increasing depth, additional laser shots result in the remelting of the material.

LIBS spectra were collected for 10 pulses (shot 1) on the first mark and the final 10 pulses (final shot) on subsequent marks during the ablation process. Figure 5 shows the Si LIBS spectra recorded on the marks as a function of the laser shots. In contrast to the result at a high repetition rate, LIBS spectra were obtained with successive laser shots (up to sixth shots), giving subsurface elemental information. However, the intensity of the Si peak decreased with an additional number of shots due to the loss of focus.

**Figure 5:**
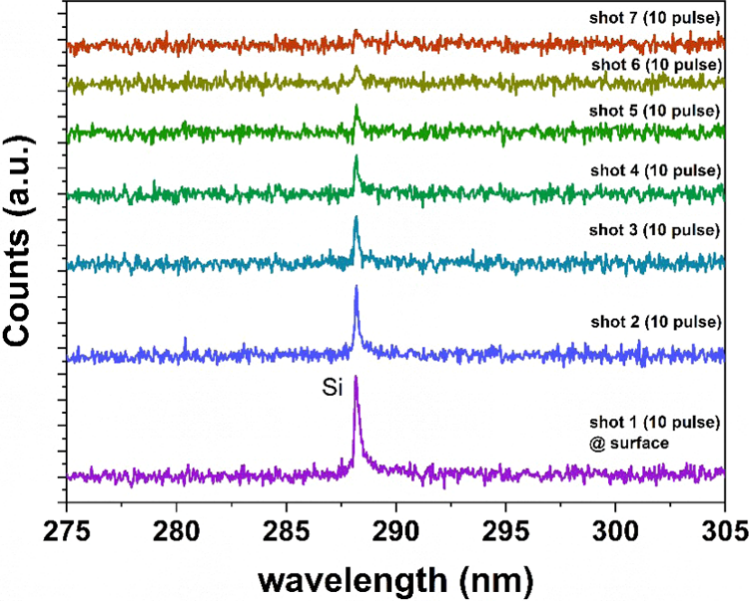
LIBS spectra of Si collected after the ablation (from marks 1 to 7 shown in Figure S9) at different laser shots.

#### 3.2.2. Pacific NW Basalt

For depth profiling of the Pacific NW basalt, the sample was first polished to reduce the surface roughness. Two different experiments, first with 10 pulses at a 1 kHz repetition rate and second with 50 pulses at a 50 Hz repetition rate, were conducted. The size and morphology of laser-ablated craters at high (1000 pulses) and low (50 pulses) pulses observed by SEM are shown in Figure 6. A higher number of laser pulses resulted in more material removal from the surface, resulting in a larger crater size (∼15 μm). In addition to material ejection, resolidification of the material around the crater walls after melting was observed. At a low number of pulses, a much smaller crater (∼10 μm) with rough walls was observed. The crater depth at 1000 pulses and 50 pulses extracted from the 3D depth profile measurement was found to be nearly 5 and 8 μm, respectively (Figure S12 in the Supporting Information).

**Figure 6:**
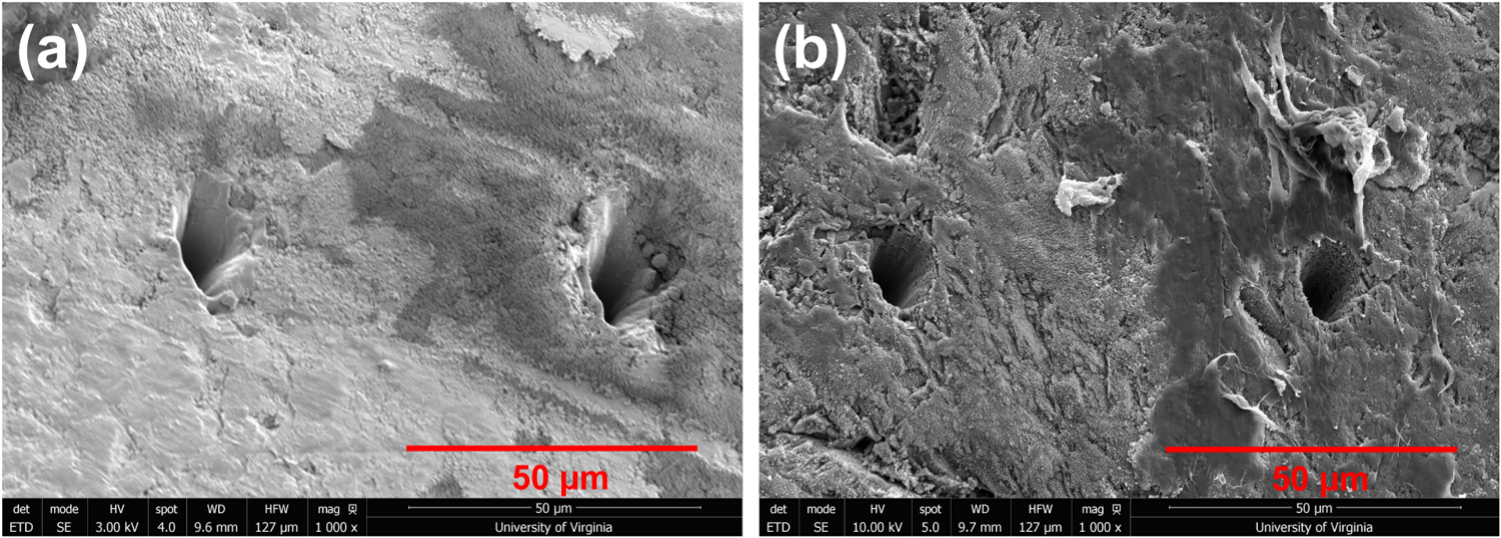
SEM image showing the crater size and morphology (a) at high (1000) and (b) low (50) number of pulses.

Figure S13a,b (in Supporting Information) show the LIBS spectra obtained for a series of laser shots with 10 and 50 pulses, respectively. The LIBS signals were collected simultaneously during the ablation process from a single point on the surface. Similar to the Si results, successive LIBS spectra showing distinct elemental peaks were obtained with additional laser shots. However, the spectra were obtained only for the first few shots, and no elemental information was obtained for a higher number of shots, thus limiting its application. To overcome the above issue, an alternative approach was considered, i.e., repeated ablation of a small area surface by scanning and performing LIBS measurement by refocusing the lens. Each scan will create a new, fresh layer of the sample, allowing a more comprehensive study of depth profiling in the sample. Figure 7 shows the LIBS spectra recorded by using this approach.

**Figure 7:**
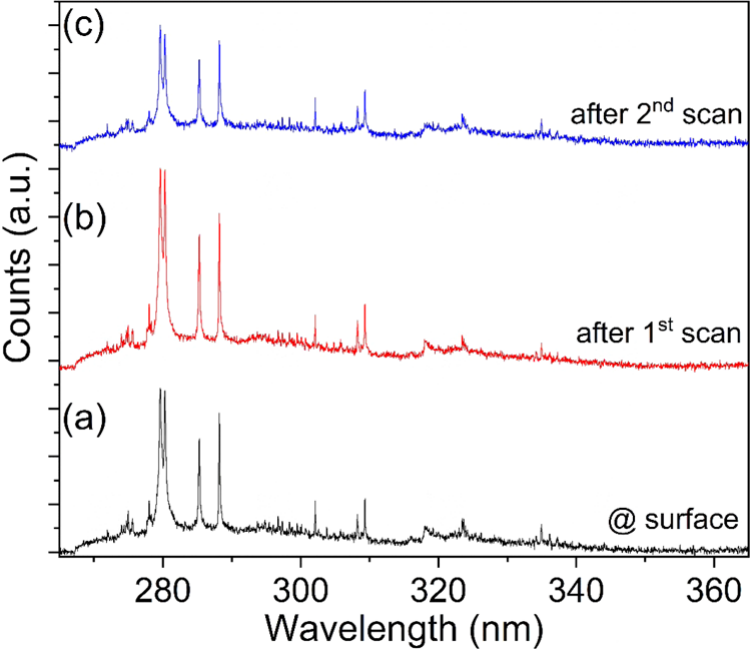
LIBS spectra recorded from a single point after repeated ablation of a small area surface (2 mm × 2 mm) by scanning: (a) At the surface, (b) after 1st scan, and (c) after 2nd scan. The acquisition time was set to 1s, and the pulse repetition rate to 1 kHz.

### 3.3 Spatially Resolved Chemical and Elemental Detection, and the Compact Size and Mass of the System

An integral aspect of the current LIBS system is its capability to detect the elemental composition at a remarkably small scale with a spatial resolution of ∼15 μm. The high spatial resolution refers to the minimum distinguishable feature size in the *x*–*y*-plane from which reliable chemical or elemental information can be obtained. To demonstrate this capability, an experiment was performed as follows. An equal proportional mixture of fine-grained powder samples of calcite and selenite minerals was prepared. This mixture was pressed into a 1 cm × 1 cm area and formed into a flat pellet. A surface image of the pellet was taken by using a high-resolution optical microscope (Hirox RH 8800), as shown in Figure 8a. The image shows a spatially uniform mixture of grains of calcite (bright grains) and selenite (dark grains), with an average grain size of ∼15 to 20 μm and a grain separation distance of about 20 μm. Three distinct spatial locations were evaluated for the Raman signal. The Figure 8b–d. show Raman signal from three different locations. In Figure 8b, measuring at location 1, the characteristic Raman peaks of selenite at 1000 and ∼3500 cm^–1^ can be observed, with minimal or no matrix interference of calcite (which shows a characteristic Raman peak at ∼1100 cm^–1^). Figure 8c shows clearly the Raman peak of calcite ∼1100 cm^–1^ at location 2, without any spatial interference of selenite. Figure 8d shows the Raman signal for location 3, where both calcite and selenite peaks are observed. These measurements confirm the system’s spatial resolution capability of 15 μm.

**Figure 8:**
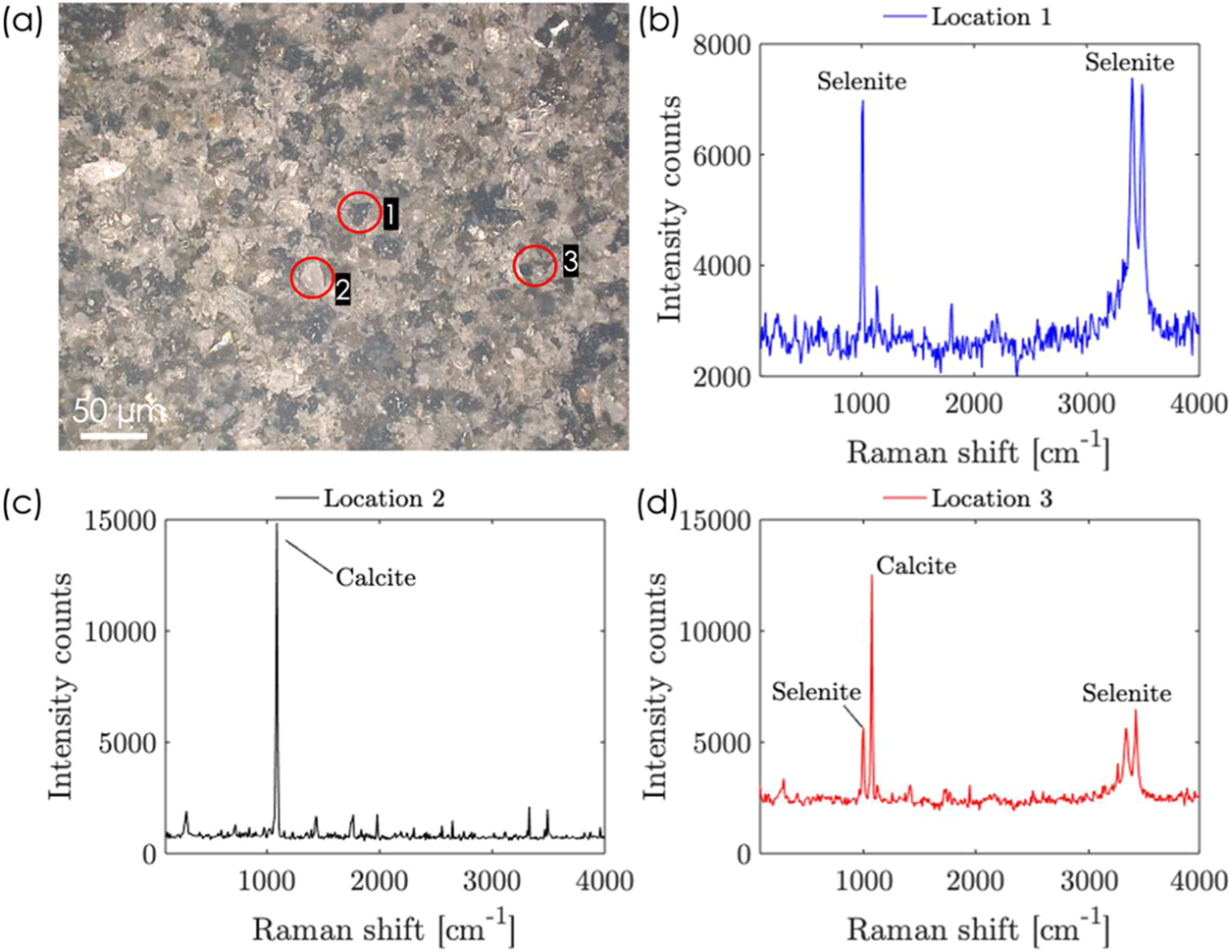
(a) Microscope image of the surface of the mixture of calcite and selenite. (b) Raman measurement at location 1, (c) location 2, (d) location 3.

The high spatial resolution becomes particularly significant when investigating potential biological signatures, as it provides a critical context for understanding the origin of organic compounds that might be present. It is worth noting that the stand-off technique employed in the SuperCam, which is a LIBS instrument used on the Perseverance rover, provides elemental information from a significantly larger ablated area with a diameter ranging from approximately 250 to 400 μm.^8^ Another attribute of the current system is its compact size and lower weight. At present, the total weight of the system (laser head, controller, and spectrometer) is about 1.67 kg. The aim is to make the system more efficient and portable by further reducing the size and weight of the optical head assembly (optics, laser, and focusing mechanism) and by the use of fiber optic bundles. The projected size, weight, and power consumption of the optical head can be around 1 cm × 1 cm × 1 cm, 20 g, and 0.5 W, respectively. This substantial reduction in weight has profound implications for mitigating the costs associated with payload delivery for space missions.

### 3.4 Fluorescence of Simulants and Geological Samples at 266 nm Wavelength

We investigated the fluorescence in both the simulant and geological field samples exposed to a 266 nm excitation wavelength. To measure the fluorescence spectra, another spectrometer from StellarNet, Inc., SILVER-Nova 25, was used to cover a wide wavelength range. Figure 9a shows the fluorescence spectra of a representative simulant. As illustrated in Figure 9a, simulant NU-LHT4M exhibited a fluorescence extending in the wavelength range of 500–900 nm, with a prominent emission peak occurring at ∼600 nm. Similar results were observed in all other simulants and geological samples. The observed peak wavelength corresponds to the higher end of the visible spectrum, with a distinct red color being perceptible in all tested samples (see the inset of Figure 9a). Results show substantial fluorescence-free regions extending up to 500 nm in the investigated samples. Additional fluorescence measurement in feldspar (Figure 9) showed a fluorescence-free region, further extending up to 600 nm. This characteristic holds a distinct advantage for Raman measurement in these samples, as it mitigates the potential for signal interference arising from fluorescence phenomena.

**Figure 9:**
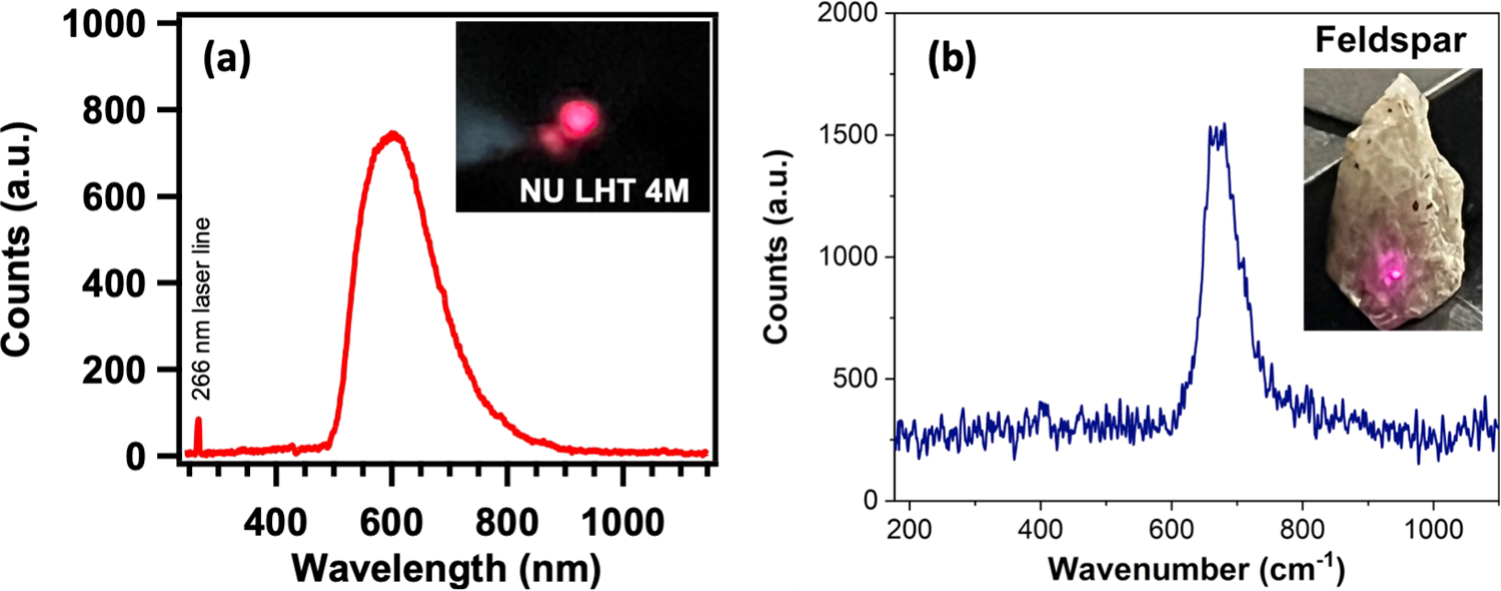
Fluorescence spectra of (a) simulant NU-LHT4M and (b) feldspar subjected to excitation at a wavelength of 266 nm. The inset shows an optical image of the emission color originating from the samples.

### 3.5 Autofocus Capabilities

The system also included the capability of maintaining autofocus during Raman and LIBS measurements on a rough surface. The system’s autofocus capability is to be able to maintain autofocus within 10 μm height variation. The system was tested for the capability of the autofocusing lens L1 by searching for the best Raman signal from a sample. The lens L1 is mounted on a translation stage, which contains a high-resolution stepping motor (Thorlabs KCH301). The stepping motor allows a micron-level translation. The laser power was attenuated, which circumvented the ablation of the sample and allowed precise autofocus without forming a crater when the beam is focused. The mineral Calcite was chosen as an example to demonstrate the autofocus capabilities. The lens L1 was moved by every 200 μm, and the peak value of the Raman spectrum was noted, as shown in Figure 10a. The maximum value occurs at 4000 μm from the initial base value and, therefore, to which the position of lens L1 is set. The spectrum recorded at 4000 μm is shown in Figure 10b, with the constituent peak of calcite at 1084 cm^–1^ being clearly shown.

**Figure 10:**
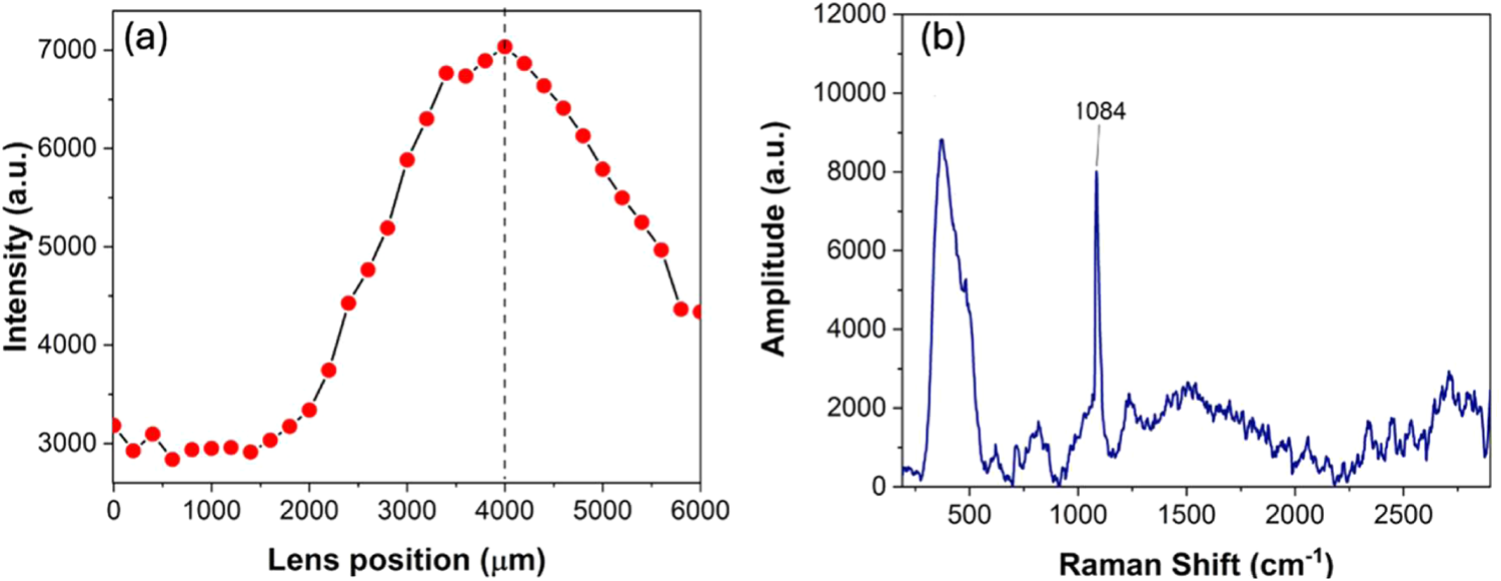
(a) Autofocus capability demonstrating the variation of the Raman signal with focus position. (b) Raman spectra of the Calcite mineral used as an example.

## 4 Discussion

Although the feasibility of utilizing a 266 nm laser for Raman mineral detection has been reported in the literature, the existing techniques often involve complex instruments, such as ICCD detectors and delay generators. Such requirements make the system more complex, bulkier, and impractical for resource-constrained planetary missions. In this paper, we have presented data on Raman measurements using 266 nm excitation in organic (alcohol, alkanes, amino acids, and polymer) and inorganic species (sulfates, carbonates), water, selenite, and calcite. The deep-UV excitation theoretically provides benefits regarding fluorescence-free Raman region, increased Rayleigh scattering (1/λ^4^), and resonance effects of organic vibrations; practical limitations arise when dealing with solid samples. In solids, strong absorption of 266 nm light leads to a shallow (nanometer scale) penetration depth, resulting in a very small number of probed molecules being subjected to Raman excitation. In addition, self-absorption of the emitted Raman signal further decreases the signal. For instance, when a 266 nm laser is used as the excitation source, it becomes challenging to detect the Raman signal in standard materials like silicon. This difficulty stems from the shallow penetration depth (∼10 nm or less) of the 266 nm laser, resulting in a lower scattering cross-section and leading to inadequate Raman signal collection.^33^ Another possibility for not acquiring Si Raman spectra is the low spectral resolution of the spectrometer, which ranges from 12.7 cm^–1^ near 0 cm^–1^ and 10.1 cm^–1^ near 4000 cm^–1^, which is greater than the full-width half-maximum (3.1 cm^–1^)^34^ of the crystalline Si Raman peak. The simulants and geological samples under study face a similar challenge of a low penetration depth of 266 nm into the material, making it difficult to acquire a Raman signal. The other contributing factor is the laser fluence. As reported in the literature, the deep-UV Raman measurements require large UV fluence (∼400 J/cm^2^),^35^ continuous sample movement, and high-sensitivity liquid nitrogen-cooled CCD detectors. The laser power density available in our case was limited to 0.4 J/cm^2^ compared to some of the reported work^35^ that used at least 59 J/cm^2^. The fluorescence measurements on these simulant and geological samples have unveiled the presence of a considerably broad fluorescence-free region extending up to 500 nm. This holds a major advantage of flexibility in laser wavelength selection for Raman measurement of such samples without worrying about the effect of fluorescence. Raman spectra of the studied samples obtained using a commercial Raman system from Renishaw at 405 nm excitation wavelength (Figure S14 in the Supporting Information) strongly corroborate the aforementioned argument.

## 5 Conclusions

In conclusion, our study demonstrated the viability of employing a 266 nm wavelength excitation source for conducting LIBS and fluorescence measurements on lunar and planetary simulants and geological field samples. We also demonstrated Raman measurement on a variety of organic and inorganic compounds and minerals, including alcohols, alkanes, amino acids, polymers, sulfates, carbonates, water, selenite, and calcite, using the LFR system at 266 nm excitation. The LFR laboratory system is characterized by its simplicity, compactness, and lightweight design (all weighing under 500 g and occupying less than 7 cm × 7 cm footprint). These features are crucial for space applications and contribute to reducing payload costs. Results affirm that precise elemental identification of both geological samples and simulants can be achieved by using a laboratory setup based on a deep-UV 266 nm laser system. Analysis of LIBS spectra revealed the presence of key elements such as magnesium (Mg), silicon (Si), iron (Fe), aluminum (Al), calcium (Ca), manganese (Mn), and titanium (Ti) in basalt, gneiss, and planetary simulants. A notable feature of the current LFR system is its capability to discern elemental compositions with micrometer-level spatial resolution (within the range of 10–20 μm). This spatial resolution enables much finer and localized elemental composition analysis and is also crucial for investigating potential biological signatures in extraterrestrial organic compounds. Furthermore, the conducted depth profiling study on Si and basalt samples using LIBS demonstrated the feasibility of achieving subsurface elemental information with a relatively low number of laser pulses (10–50 pulses), as opposed to requiring a higher pulse count. Nonetheless, it is important to acknowledge that while spectroscopic data was successfully captured during the initial series of laser shots, subsequent iterations involving a larger number of shots could not yield any elemental information. An alternative approach to depth profiling, i.e., the area scan technique, is proposed, which has proven to be more effective than the single spot ablation method in these samples. Additionally, the fluorescence measurements in these samples showed a wide fluorescence-free region from 266 to 500 nm, suitable for Raman measurements. An additional study based on performing sensitive detection of mixtures is made in refs 36–38 . These findings hold significant relevance for advancing LFR instruments in the realm of planetary science missions, particularly in scenarios where mass, volume, power consumption, and cost are constrained.

## Supplementary Material



## Data Availability

All of the data is available throughout the manuscript and the supporting.

## Notes

Comparative data on LIBS emission lines for standard elements, LIBS, Raman, and fluorescence spectra using 266, 405, and 1064 nm excitation for comparative purposes. Optical images of geological samples, SEM/EDS images, and depth profiling 3D images, all originally taken by the authors (PDF)
