# Supplementary material for: A High Spatial and Depth Resolution Deep-UV 266 nm Wavelength Laser-Based Integrated LIBS, Fluorescence, and Raman System for Probing Lunar and Planetary Simulants and Geological Materials
Source: ACS Omega. 2025 Aug 15;10(36):40958–67. doi: 10.1021/acsomega.5c02748 (PMC12444541; doi:10.1021/acsomega.5c02748)
Supplement: Supplementary file 1 [file ao5c02748_si_001.pdf]

A High Spatial and Depth Resolution Deep-UV 266 nm Wavelength Laser-based Integrated LIBS, Fluorescence, and Raman System for Probing Lunar and Planetary Simulants and Geological Materials

Anil Aryal[1,2*], Pawan K. Kanaujia[1], Atchutananda Surampudi[1], Dina M. Bower[3,4], Tilak Hewagama[4], Narasimha S. Prasad[5], William B. Moore[6,] and Mool C. Gupta[1*]

[1] *Charles L. Brown Department of Electrical and Computer Engineering, University of Virginia, Charlottesville, VA, 22904, USA*

[2] *Laser and Plasma Technologies, Charlottesville, VA, 22904, USA*

[3] *University of Maryland, Department of Astronomy, College Park, MD 20742, USA*

[4] *NASA Goddard Space Flight Center, Greenbelt, MD 20771, USA*

[5] *NASA Langley Research Center, Hampton, VA 2368, USA*

[6] *Department of Atmospheric & Planetary Sciences, Hampton University, Hampton, VA, 23668, USA*

SUPPLEMENTAL MATERIALS

Table S1: List of the emission lines observed in the LIBS spectrum of common elements compared with the NIST database.[1]

Material	NIST (nm)	Measured (nm)	Difference (nm)
Si	288.16	288.2	0.04
	298.76	298.8	0.04
Sn	270.65	270.69	0.04
	277.98	277.99	0.01

	283.99	284.01	0.02
	285.06	285.05	0.01
	286.33	286.36	0.03
	300.91	300.93	0.02
	303.41	303.45	0.04
	317.5	317.51	0.01
	326.23	326.24	0.01
	333.06	333.07	0.01
Ge	269.13	269.18	0.05
	270.96	271	0.04
	275.46	275.48	0.02
	303.9	303.89	0.01
	312.48	312.5	0.02
	326.95	326.97	0.02
Al	281.62	281.67	0.05
	308.22	308.25	0.03
	309.27	309.35	0.08
Ti	308.8	308.83	0.03
	323.45	323.5	0.05
	334.94	334.9	0.04

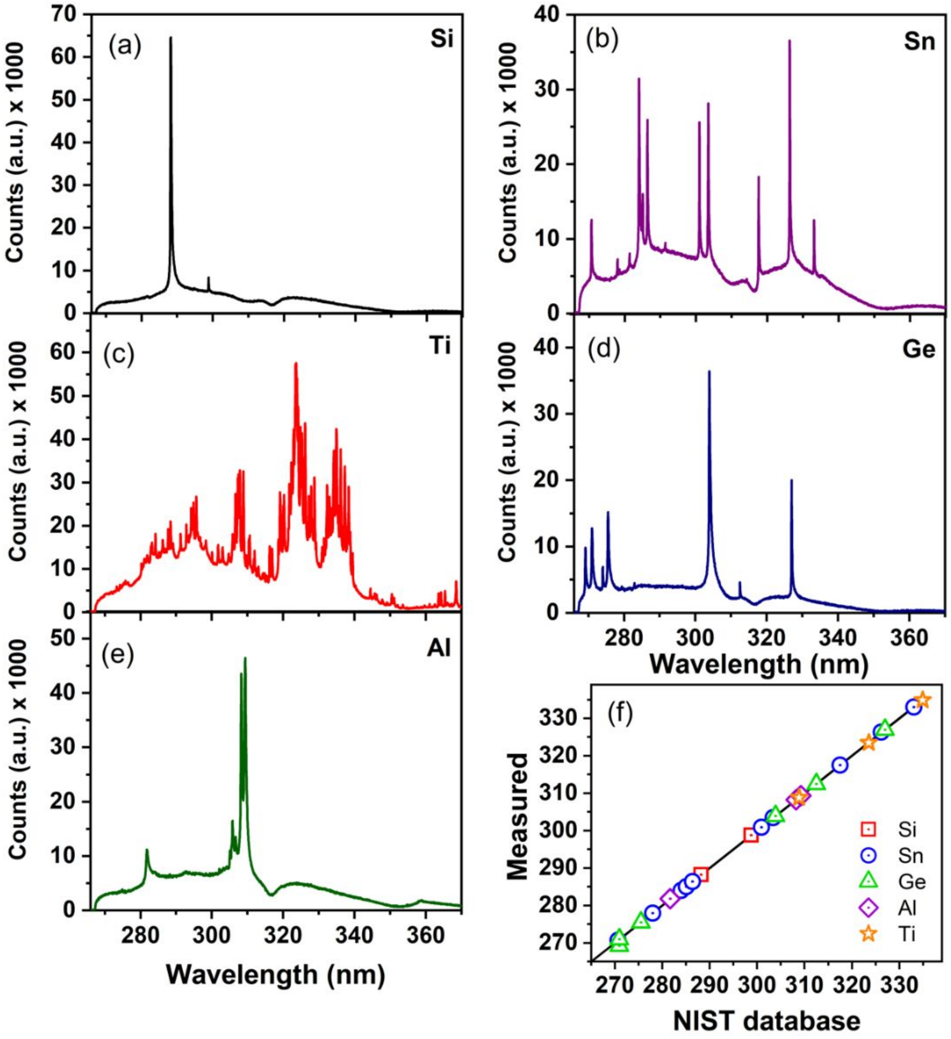

Figure S1: (a)-(e) Measured LIBS spectra of common planetary materials. Several sharp peaks on the LIBS spectra are attributed to the ionic emission from these materials. (f) Comparison of LIBS elemental peak positions with NIST database.[1]

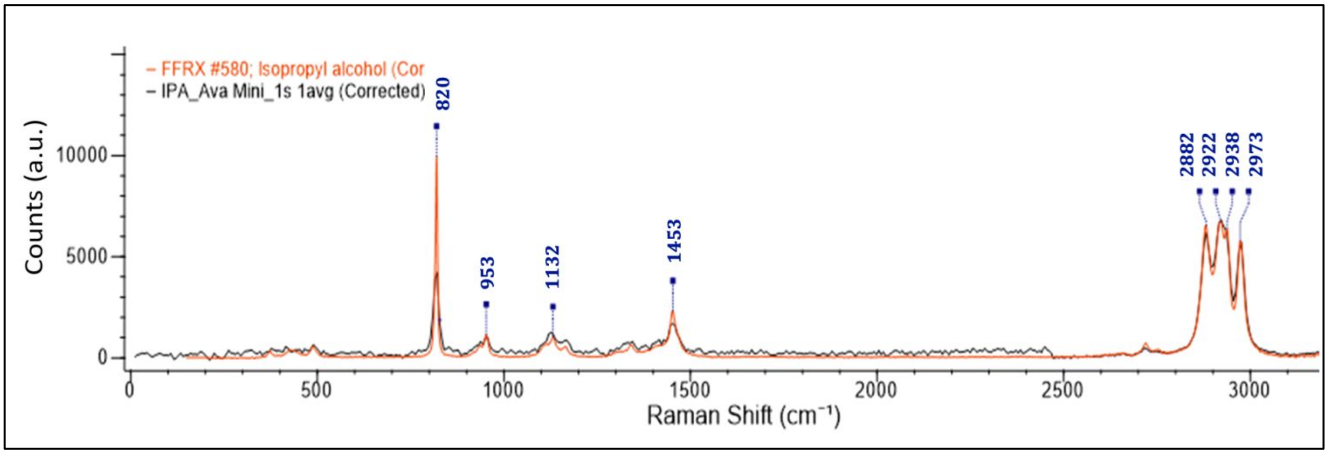

Figure S2: Measured Raman Spectra of the IPA obtained using a 266 nm excitation source showing good agreement with known peak positions. The black spectrum represents the measured data, while the red spectrum represents the spectral match with the database using "*Know it All*" software.

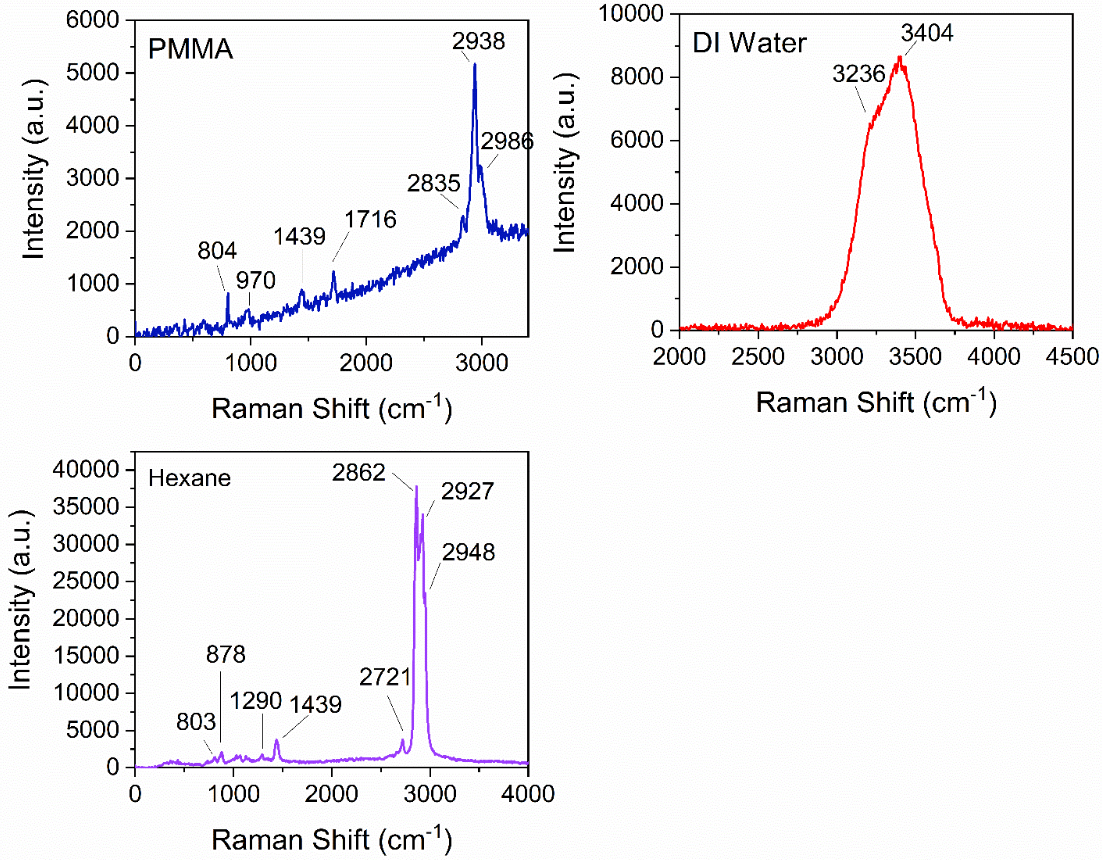

Figure S3: The measured Raman spectra of PMMA, DI water, and hexane.

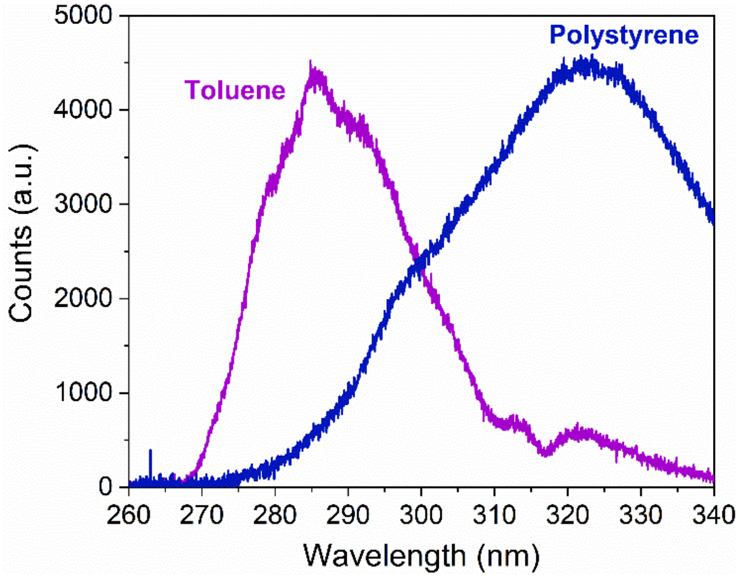

Figure S4: Measured fluorescence emission spectra of toluene and polystyrene under 266 nm excitation wavelength.

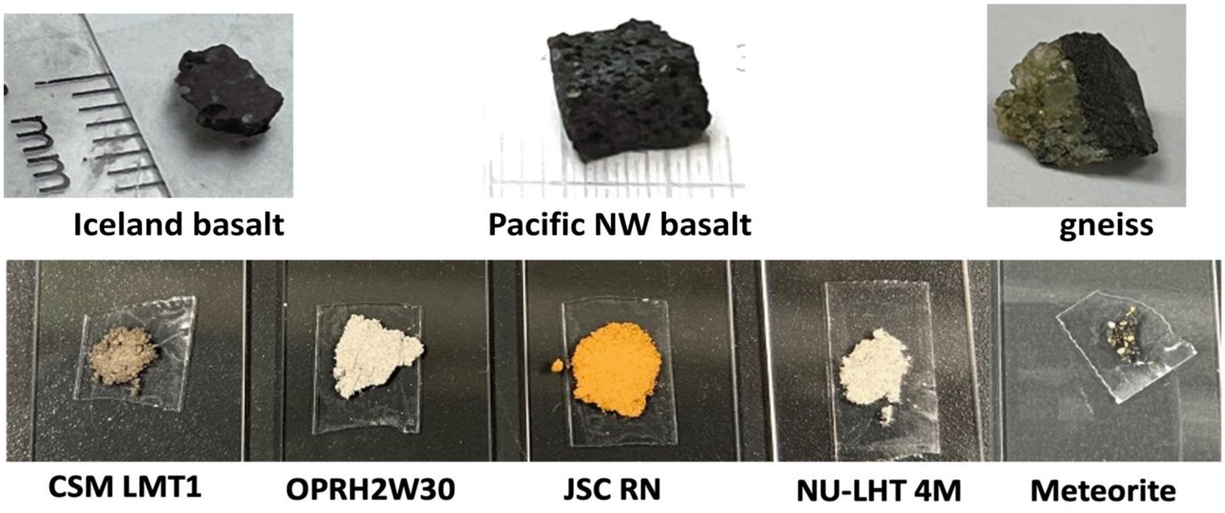

Figure S5: Images of geological samples (upper panel), lunar and planetary simulants, and a meteorite (lower panel), used in this study. The simulants are in powder form.

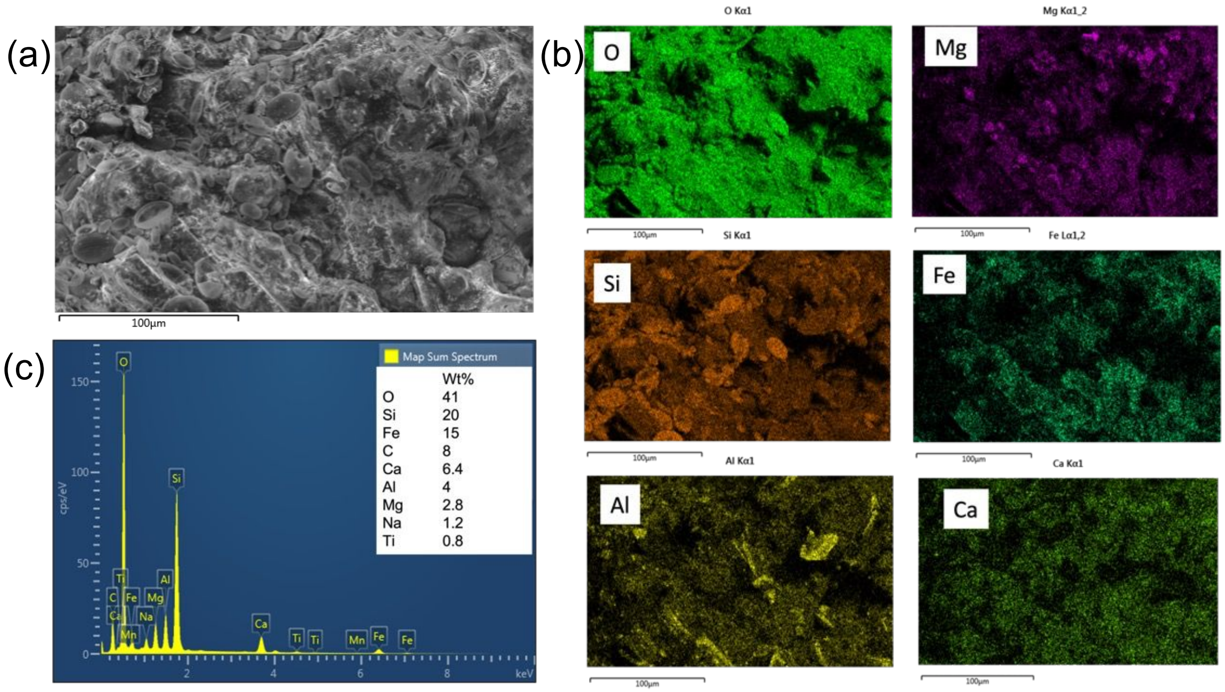

Figure S6: Measured data (a) SEM image, (b) EDS mapping, and (c) EDS spectrum of Iceland basalt.

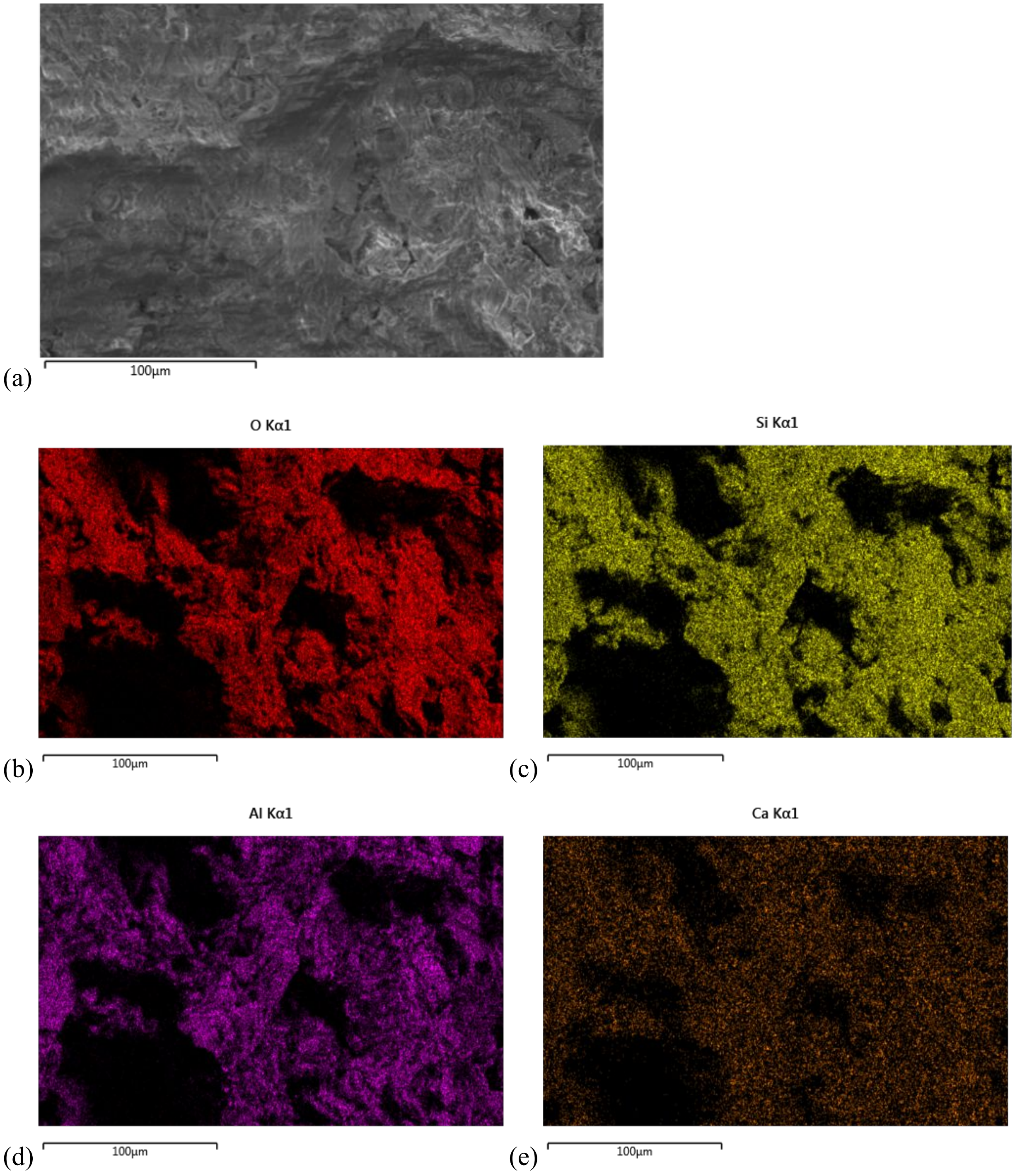
(a)
100µm
O Kα1
Si Kα1
(b)
100µm
(c)
100µm
Al Kα1
Ca Kα1
(d)
100µm
(e)
100µm

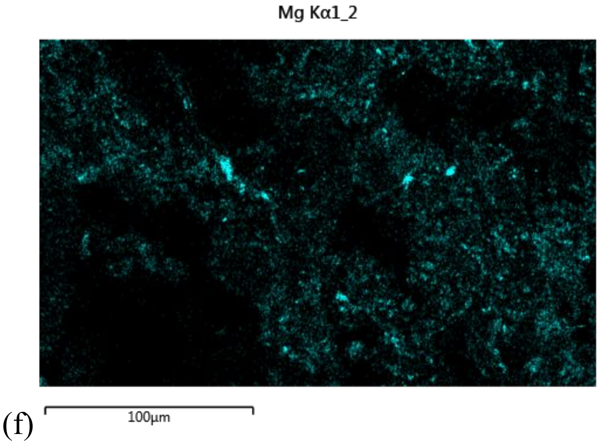

(f)

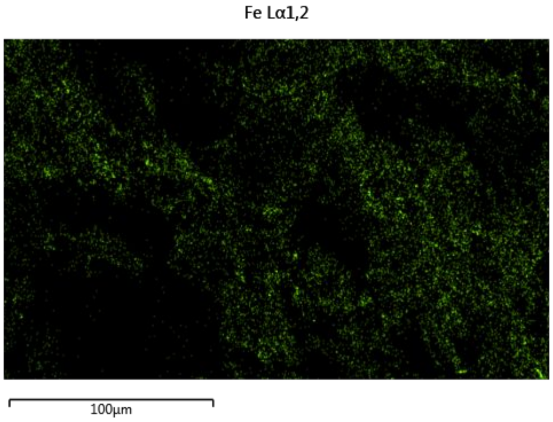

(g)

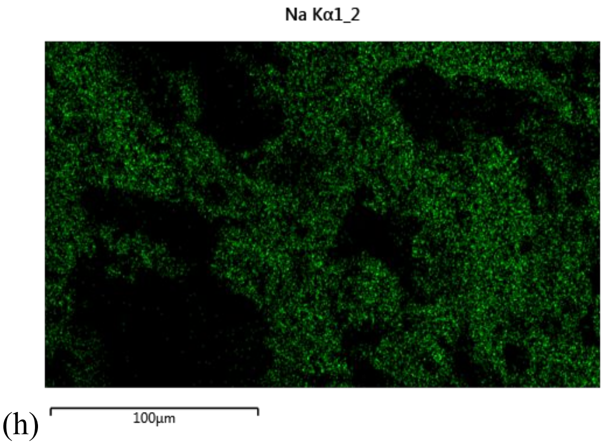

(h)

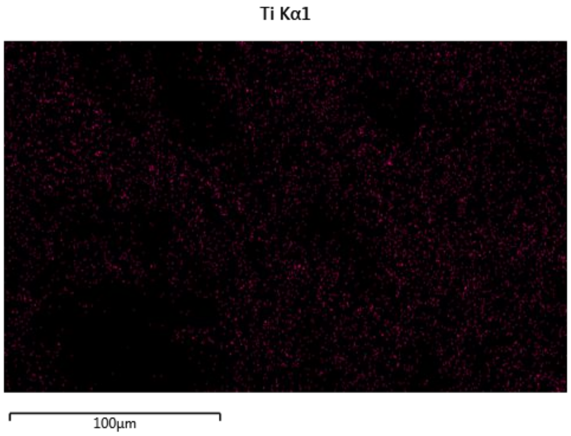

(i)

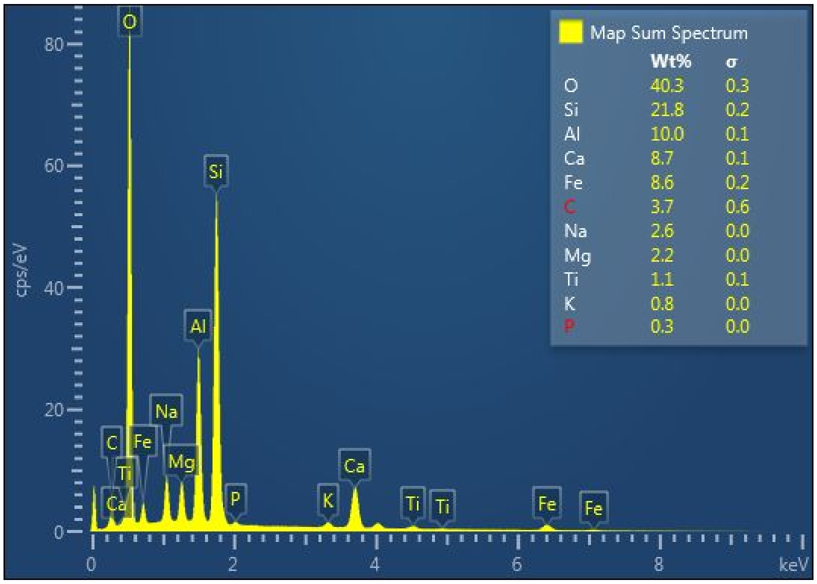

(j)

Figure S7 Measured data: (a) the SEM image, (b)-(i) EDS mapping, and (j) EDS spectrum of JSC-1 simulant.

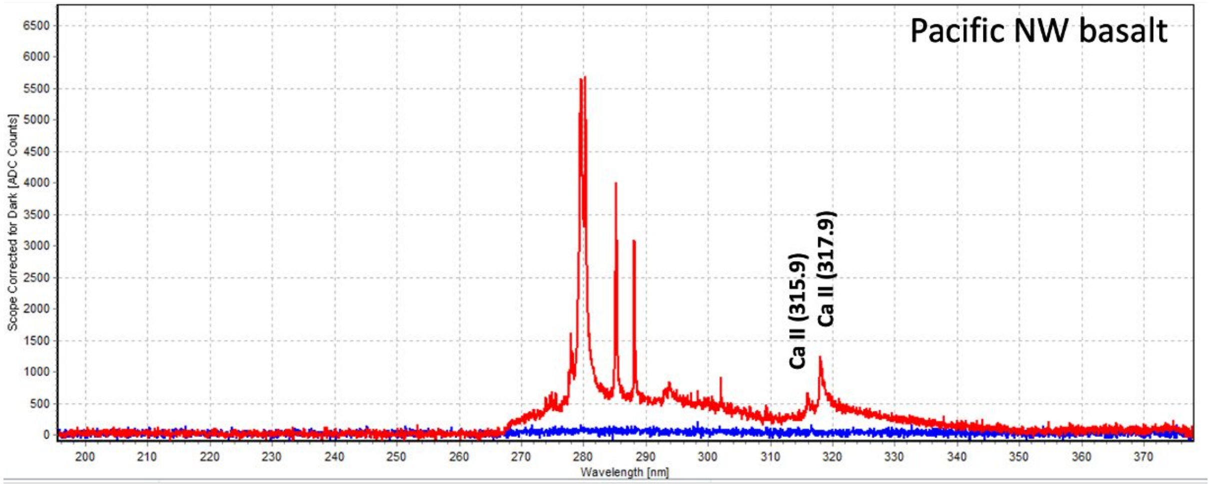

Figure S8: LIBS spectra of Pacific NW basalt recorded during the first shot at the surface (spectra in red).

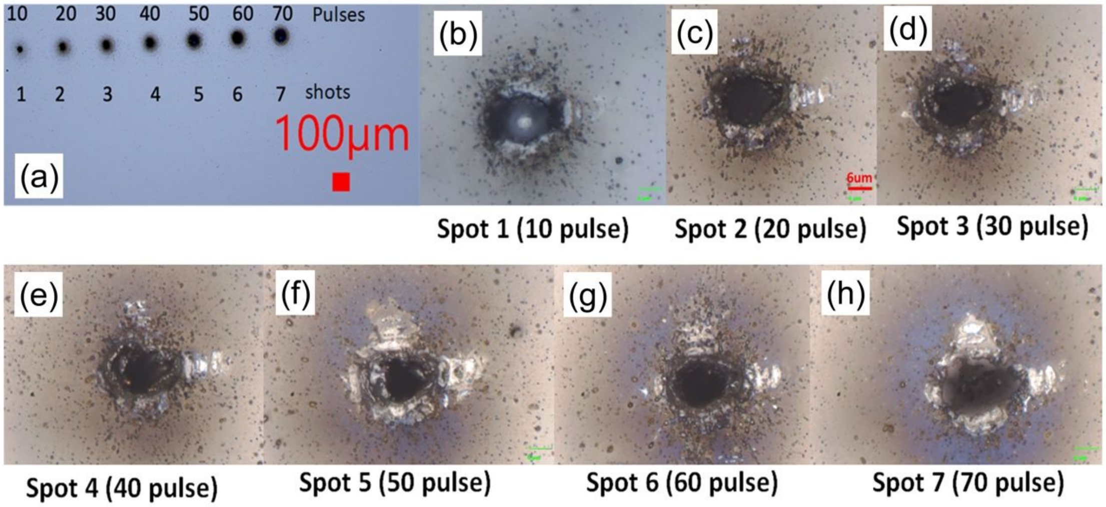

Figure S9: (a) Series of laser marks 1 through 7 ablated on Si wafer (b)-(h) 2D magnified images of the craters formed by ablation.

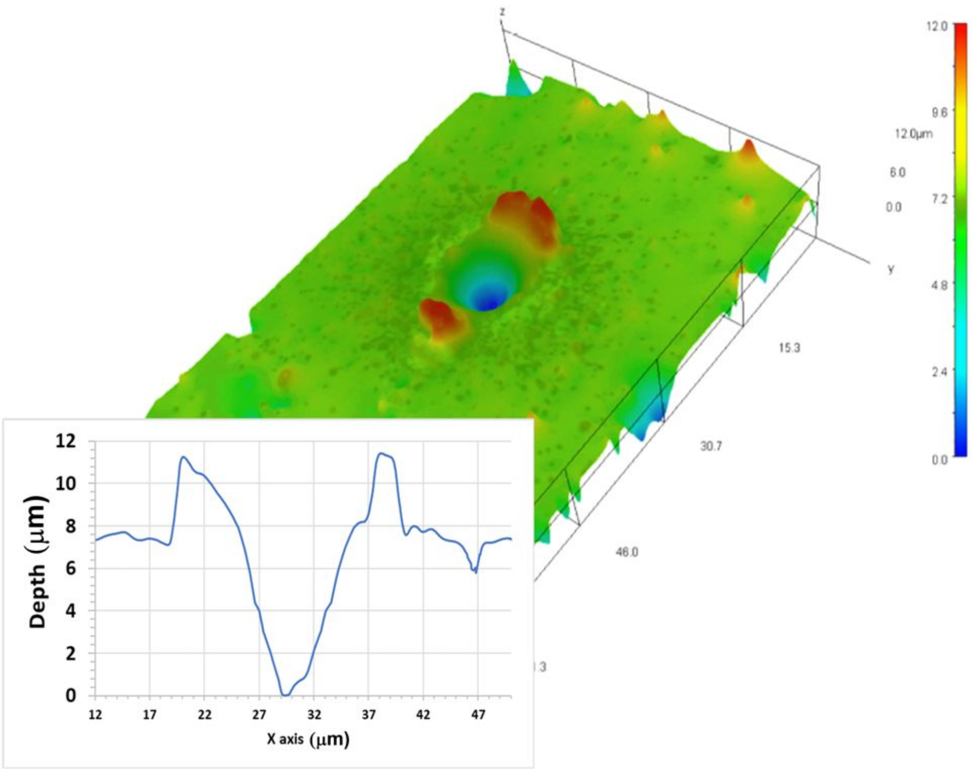

Figure S10: 3D topographic image profile of the crater formed by the first set of 10 pulses at the Si surface in Figure S9. Inset: depth profile of the crater formed.

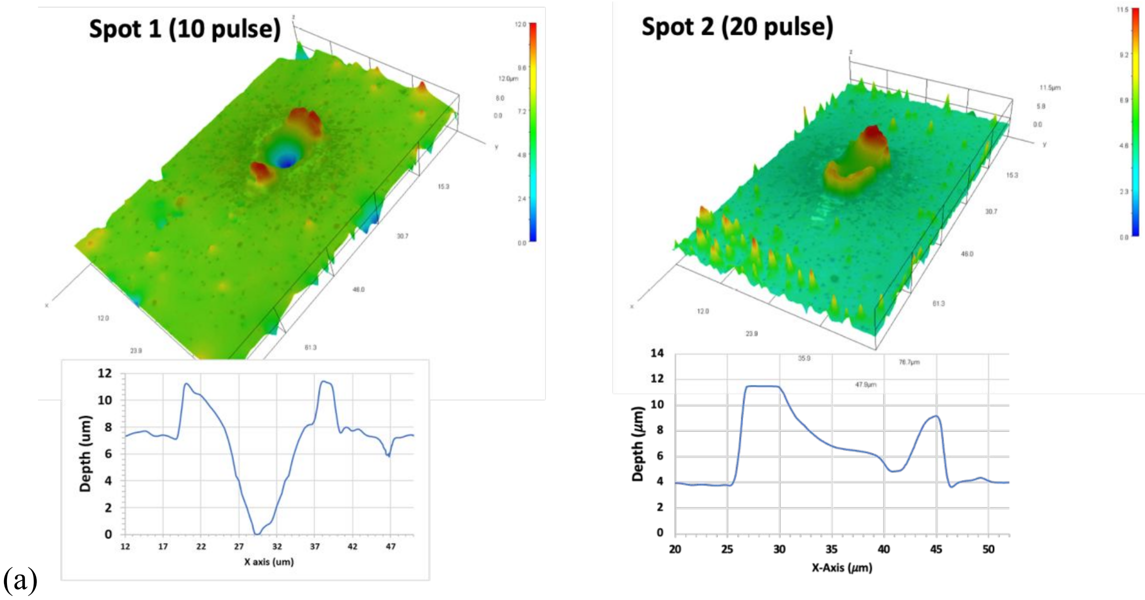

(a)

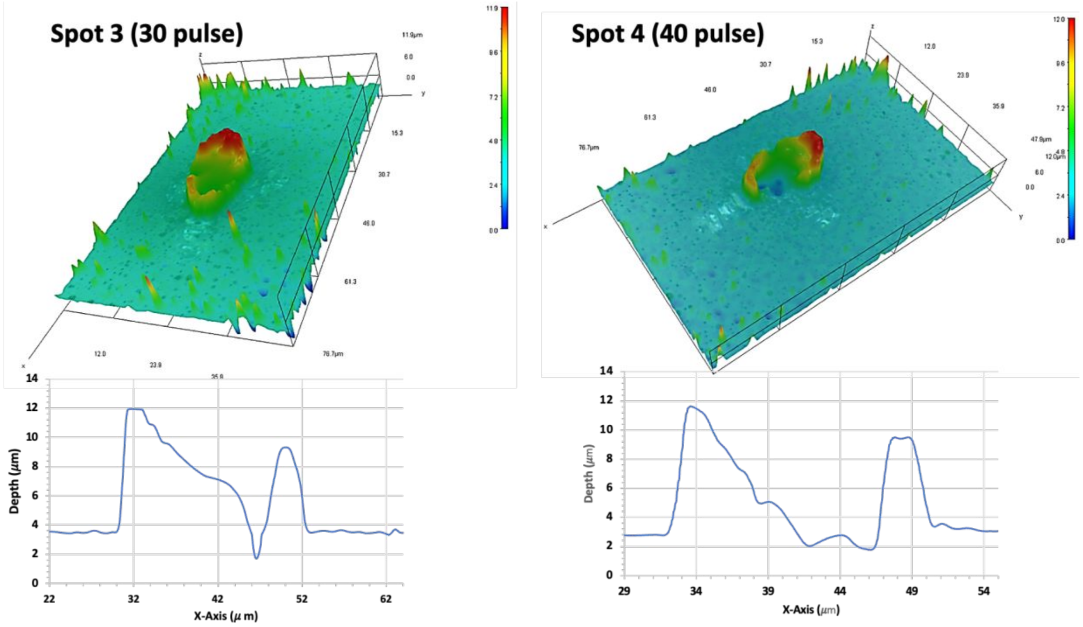
Spot 3 (30 pulse)
Spot 4 (40 pulse)
Depth (μm)
X-Axis (μ m)
Depth (μm)
X-Axis (μm)

(b)

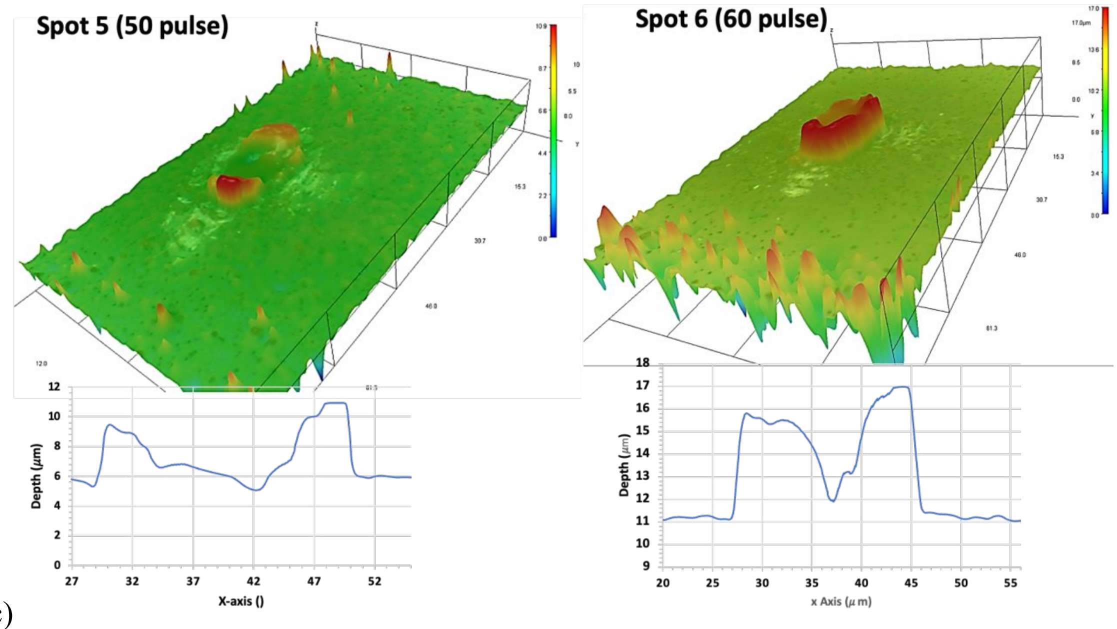
Spot 5 (50 pulse)
Spot 6 (60 pulse)
Depth (μm)
X-axis ()
Depth (μm)
x Axis (μ m)

(c)

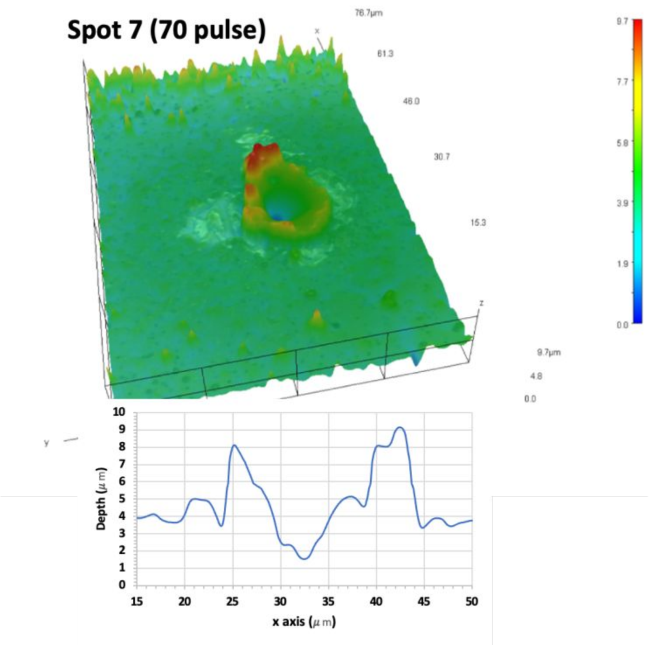

(d)

Figure S11: (a)-(d) Measured 3D topographic image and depth profile of craters in Si shown in Figure S9.

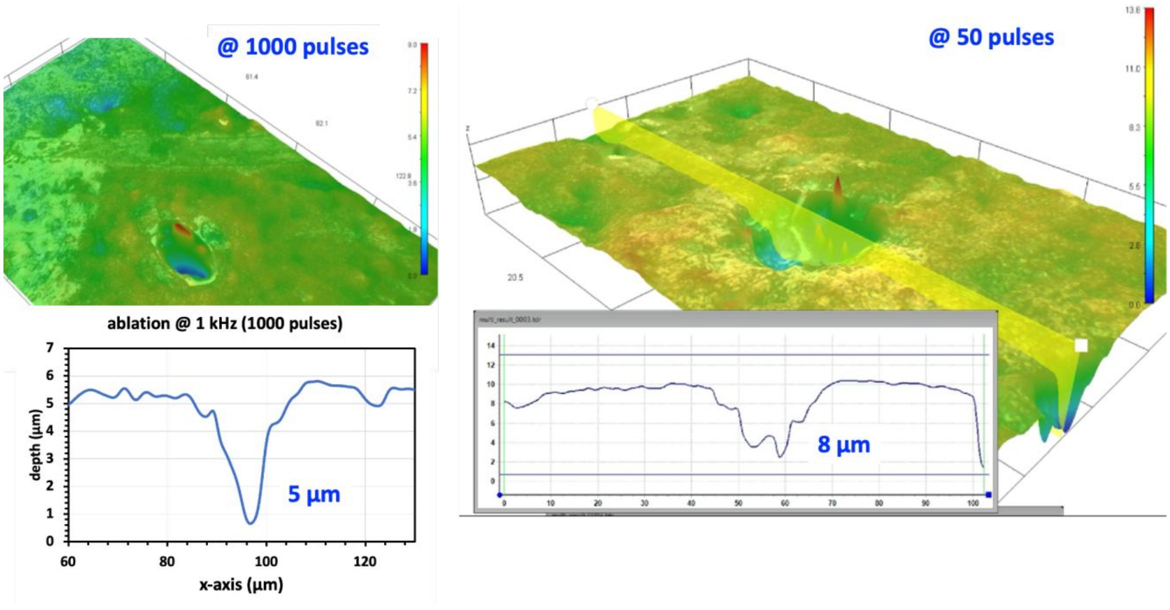

Figure S12: 3D topographic image and depth profile of craters formed in Pacific NW basalt at 1000 and 50 pulses.

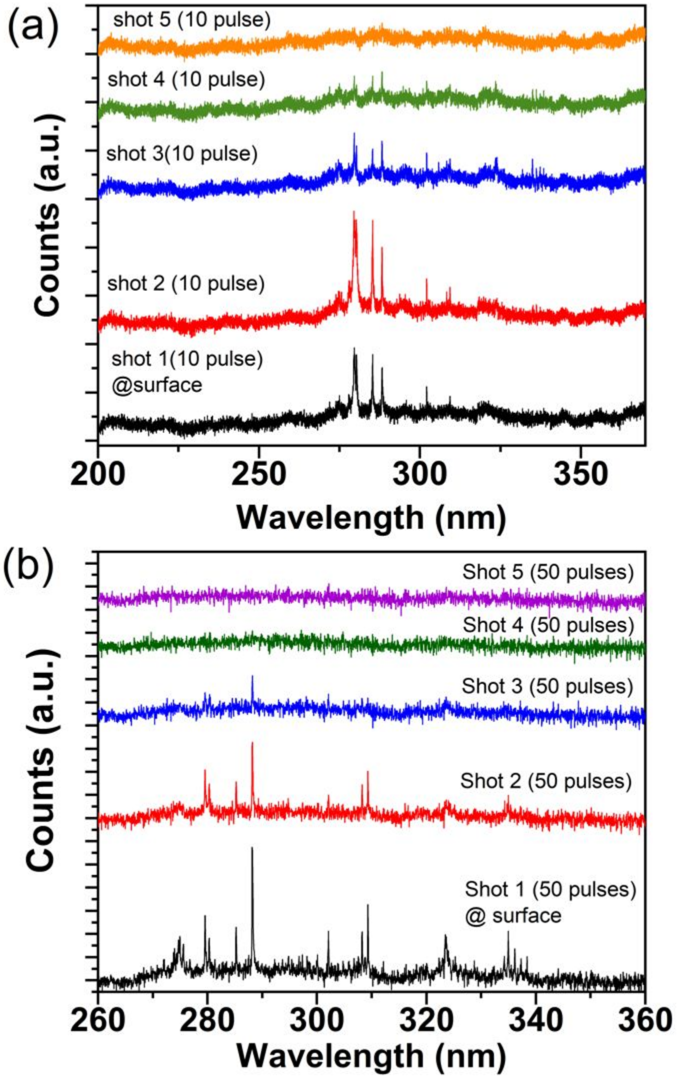

Figure S13: LIBS spectra obtained for a series of laser shots with (a) 10 pulses and (b) 50 pulses.

Depth of focus (DOF) of lens L1:

DOF = $\eta \frac{\lambda}{2\,(N.A.)^2}$ ...... (1)

where, η = refractive index of the medium (air =1)

N. A. = numerical aperture of objective = 0.28

λ = wavelength of light (266 nm in our case)

The DOF of lens L1 = 1.7 µm, which is less than the depth of the crater formed (5 to 8 µm)

Theoretical estimation of change in power density of laser from focus

Focal length (f) = 20 mm

N.A. = 0.29

Diameter of lens L1 (ϕ) = 7.4 mm

Half angle (θ) = $\sin^{-1}$ (N.A) = 16.26°

The diameter of the focal spot can be calculated from Rayleigh criterion for the diffraction limit of the Airy spot as,

$2\,r_o = 1.22\frac{\lambda}{N.A.}$.

$2\,r_o$ = 1.11 µm

Let z = 0 be the position of the focal plane in z-direction.

Considering the shape of the longitudinal section of the focused laser beam described by the hyperbola,

$$\frac{x^2}{a^2} - \frac{z^2}{b^2} = 1$$

For a = $r_0 \approx$ 0.56 µm

b = r_0 tan (90 - θ) = 1.85 µm

$$\frac{x^2}{{r_0}^2} = 1 + \frac{z^2}{b^2}$$

The ratio $\frac{x^2}{{r_0}^2}$ helps describe how the power density changes as the radial distance from the center of the beam (x) varies relative to the initial radius of the spot (r_0).

Change in power density as a function of the shift from focal point z = 0 to 8 μm (depth of crater) is $\frac{x^2}{{r_0}^2} = 1 + \frac{z^2}{b^2} = 1 + \left(\frac{8}{1.85}\right)^2 \sim 19.7$

This shows the power density of the exciting laser light changes by ~ 20 times the magnitude at focus when moved to 8 μm.

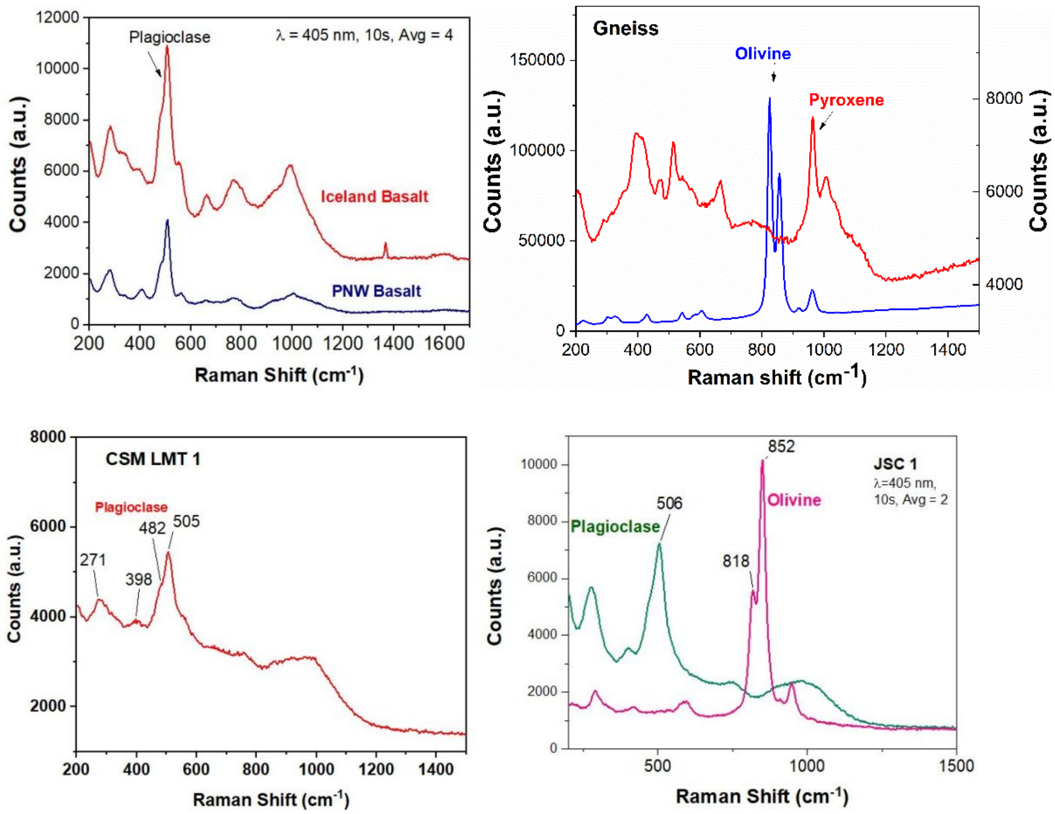

Figure S14: Raman spectra of the studied samples obtained using a commercial Raman system from Renishaw at 405 nm excitation wavelength.

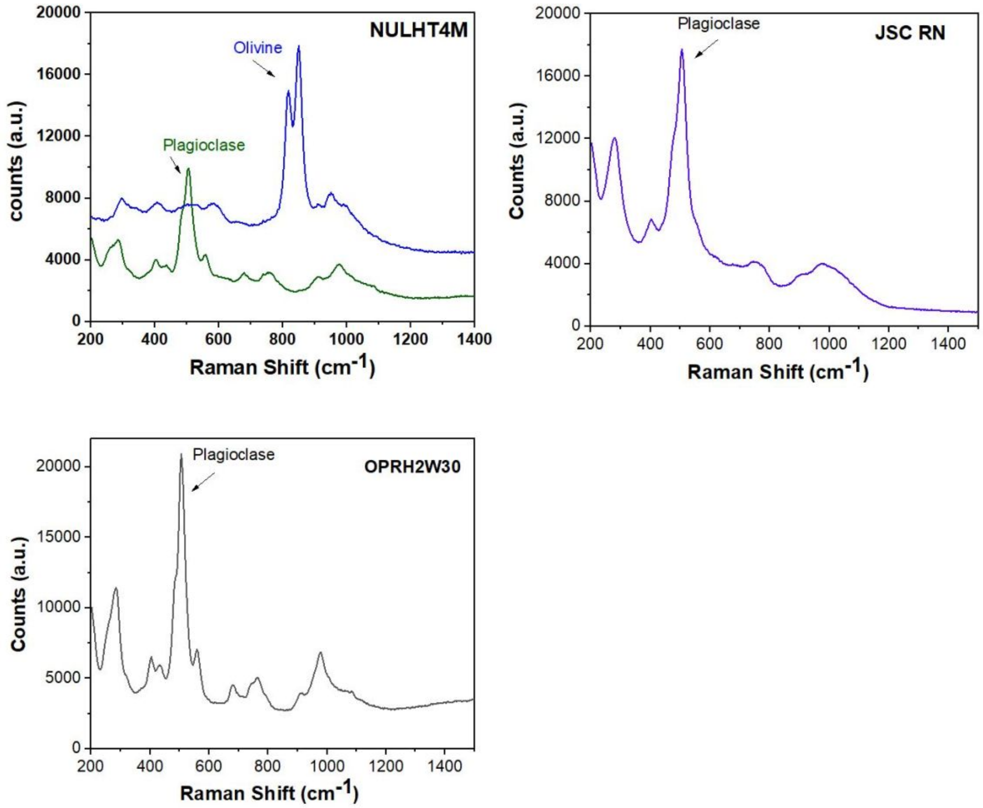

Figure S15: Raman spectra of the studied samples obtained using a commercial Raman system from Renishaw at 405 nm excitation wavelength.

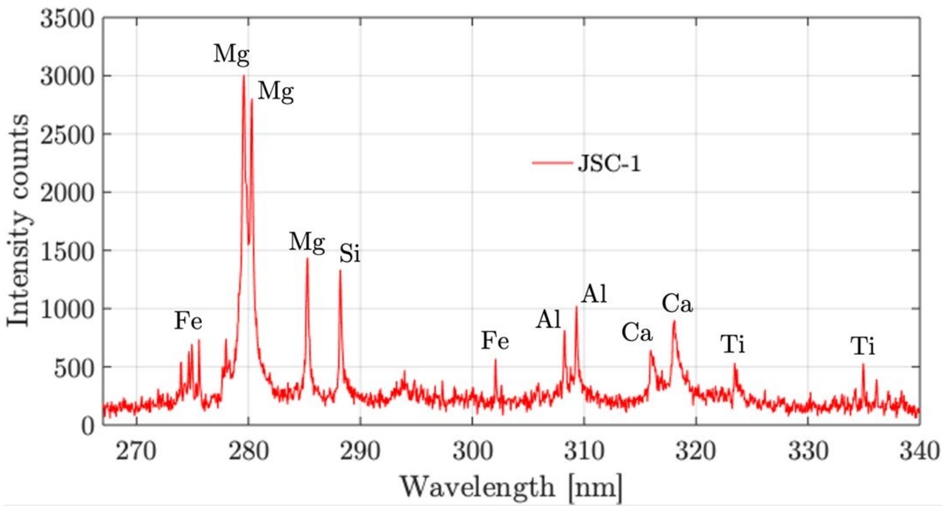

Figure S16. LIBS spectra of JSC-1 simulant measured using a 1064 nm laser-based LIBS system.

References

1. National Institute of Standards and Technology, https://physics.nist.gov/PhysRefData/ASD/LIBS/libs-form.html, accessed May 2023.

2. Paweł Borowicz. "Depth-Sensitive Raman Investigation of Metal-Oxide-Semiconductor Structures: Absorption as a Tool for Variation of Exciting Light Penetration Depth", Journal of Spectroscopy. 2016. Volume 2016:1-14. https://doi.org/10.1155/2016/1617063.
